# Polymeric Nanotubes as Drug Delivery Vectors—Comparison of Covalently and Supramolecularly Assembled Constructs

**DOI:** 10.1021/acs.biomac.2c00063

**Published:** 2022-05-18

**Authors:** Andrew Kerr, Erny Sagita, Edward D. H. Mansfield, Tri-Hung Nguyen, Orlagh M. Feeney, Colin W. Pouton, Christopher J.H. Porter, Joaquin Sanchis, Sébastien Perrier

**Affiliations:** † Department of Chemistry, The University of Warwick, Coventry CV4 7AL, U.K.; ‡ Drug Delivery Disposition and Dynamics, Monash Institute of Pharmaceutical Sciences, Monash University, 381 Royal Parade, Parkville 3052, VIC, Australia; § Warwick Medical School, The University of Warwick, Coventry CV4 7AL, U.K.

## Abstract

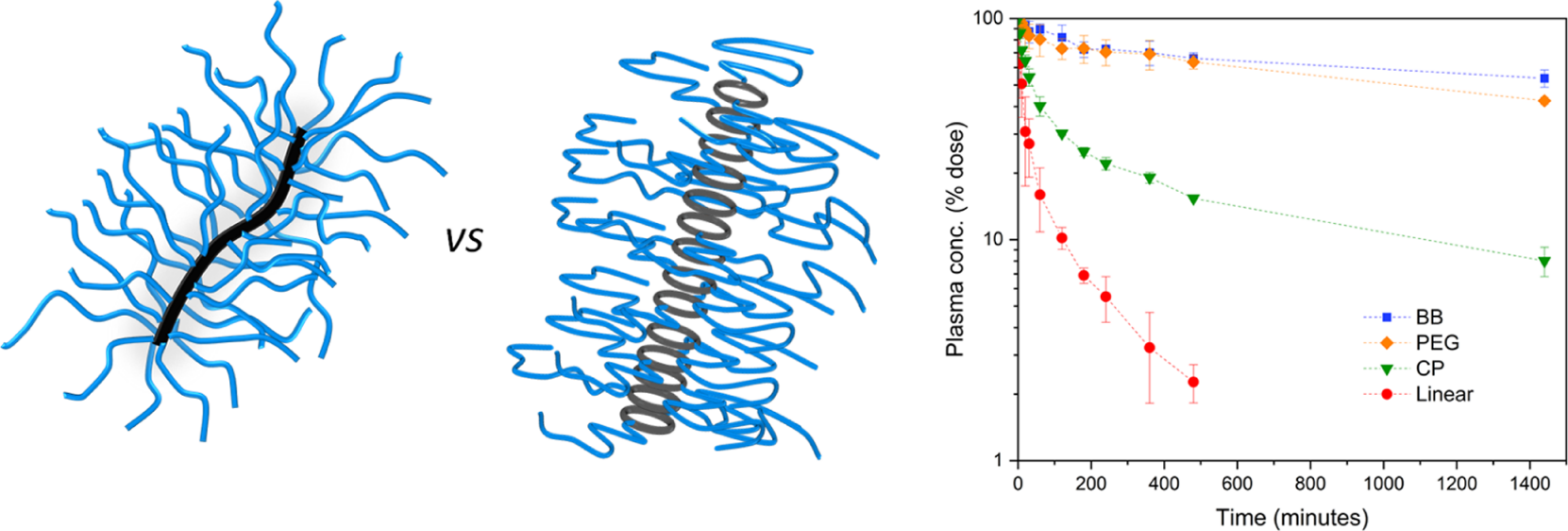

Rod-shaped nanoparticles have been identified as promising drug delivery candidates. In this report, the in vitro cell uptake and in vivo pharmacokinetic/bio-distribution behavior of molecular bottle-brush (BB) and cyclic peptide self-assembled nanotubes were studied in the size range of 36–41 nm in length. It was found that BB possessed the longest plasma circulation time (*t*_1\2_ > 35 h), with the cyclic peptide system displaying an intermediate half-life (14.6 h), although still substantially elevated over a non-assembling linear control (2.7 h). The covalently bound BB underwent substantial distribution into the liver, whereas the cyclic peptide nanotube was able to mostly circumvent organ accumulation, highlighting the advantage of the inherent degradability of the cyclic peptide systems through their reversible aggregation of hydrogen bonding core units.

## Introduction

The use of nanoparticle technologies to deliver pharmaceutical actives to the body in a controlled manner has received intense scientific focus over the last few decades.^1^ In addition to the type of nanomaterial used as the delivery vector (e.g., dendrimers,^2^ liposomes,^3^ inorganic nanoparticles,^4^ polymeric assemblies,^5^ and nanogels^6^), nanoparticle morphology has been established as an important parameter for controlling properties in terms of circulation time, cell association, and tumor penetration.^7−9^ Spherical nanoparticles have been most frequently studied, primarily due to the ease of synthesis; however, discs,^10^ rods,^11,12^ filomicelles,^13^ and nanoneedles^14^ have also been investigated. Among the array of studied morphologies, rod-shaped nanoparticles stand out as possessing excellent potential,^15^ for example, when compared to their similarly sized spherical counterparts, rod-shaped silica nanoparticles displayed increased rate and concentration of accumulation in tumors.^16^ The difference was rationalized by the reduced dimension of tubes in one dimension, improving the ability to permeate through pores, which was corroborated by an increased permeation in vitro through collagen gels. Polystyrene rod-shaped particles also showed higher cellular uptake than spherical/disk morphologies,^17^ and pharmacokinetic analysis of a poly(ethylene glycol) (PEG)-coated tobacco mosaic virus nanotube revealed increased circulation times compared to a spherical equivalent.^18^

Bottle-brush (BB) macromolecules are an enticing option as drug delivery vectors as the ability to precisely tune dimensions by modification of the backbone and side-chain degree of polymerization allows for facile synthesis of a range of rod-shaped particles and therefore provides an effective platform for the investigation of morphological effects.^19^ Existing as a unimolecular species, they do not rely on self-assembly as per worm-like polymer micelles, and chemical functionalization can be performed at selective positions among the backbone, side chains, or end groups of the macromolecule. BBs have been studied for use as degradable polymer–drug conjugates and as MRI contrast agents.^20−22^ PEG-based BBs of varying aspect ratios were studied in vivo,^23^ where the longer materials possessed similar blood circulation times to shorter BBs but displayed increased organ accumulation. A BB of the aspect ratio of ∼4–5 was found to give the highest penetration into spheroid models when compared to spherical or filamentous counterparts,^24^ and in further work, it was found that the BB stiffness is also an important parameter, whereby particles of high stiffness showed preferential cellular localization into the mitochondria.^25^

One challenge with designing effective delivery systems is potential accumulation in the body,^26^ which can be mitigated by the incorporation of biodegradable linkages into polymeric materials, allowing them to be broken down into low-molecular-weight (MW) fragments. Such an approach has been incorporated into the design of several brush-like systems, including nanoworms,^27^ brush star-arm polymers,^28^ and side-chain or backbone degradable BBs.^20,29^ Alternatively, the synthesis of nanoparticles through a bottom-up supramolecular self-assembly approach leads to a system with inherent degradability, potentially circumventing toxic long-term accumulation of vector through the gradual disassembly of the nanotube into small, readily cleared unimers. An example of such a system is cyclic peptide (CP) consisting of an even number of alternating D and L amino acids, which assemble into nanotubular structures driven through directional hydrogen bonding interactions of the amide groups.^30^ Cyclic peptides have seen use in a number of biological applications. A range of 6–8 unit cyclic peptides displayed high activities against *Staphylococcus aureus* and *Escherichia coli* in vitro and are believed to act through interactions with the bacterial membrane, increasing permeability and disrupting ion potentials.^31,32^ This membrane interaction behavior was exploited for drug delivery whereby the cellular uptake of small molecule anticancer drugs was significantly enhanced in the presence of cyclic peptides, leading to the reduced IC50 values.^33^

Cyclic peptides, however, tend to possess poor solubility in aqueous solution due to a tendency to form large aggregates and can therefore be challenging to adapt as a drug delivery vectors directly. Modification of the cyclic peptides with polymeric side chains is an effective technique for controlling the material properties, allowing for improvement of solubility, a degree of control of the size of assembly by altering polymer sterics, while also providing a handle for introducing further functionalities.^34−36^ An initial study on the pharmacokinetic properties of cyclic peptide poly(hydroxypropyl methacrylamide) conjugates demonstrated an extended plasma circulation time when compared to a linear polymer of equivalent MW.^37^ Janus nanotubes can be accessed by conjugation of two different polymeric arms to the peptide core,^38^ leading to further self-assembly into thicker bundles of nanotubes. Amphiphilic variants of such structures were shown to interact with large unilamellar vesicles to release an entrapped dye, whereas the janus nanotubes were able to induce escape of small molecules in vitro from the lysosomal compartment.^39^

Conjugation with PEG has been extensively used to modify nanoparticles, drugs, and proteins,^40,41^ in order to increase circulation times by imparting a stealth effect to avoid immune system response.^42,43^ There have been increasing concerns, however, of an accelerated blood clearance (ABC) effect in PEG materials, whereby subsequent administrations of the formulation invoke an immune response, leading to dramatically decreased circulation times,^44^ believed to result from the production of anti-PEG immunoglobulin M antibodies.^45^ Polyacrylamides are a promising class of water-soluble materials, which typically have low toxicity and some degree of stealth effect in vivo, with initial studies revealing they do not display an ABC phenomenon and thus may be suitable as alternatives to PEG in biomedical applications.^46,47^ Of this monomer family, poly(*N*-acryloylmorpholine) (PNAM) is particularly appealing due to its high solubility in water and a variety of organic solvents and its well-controlled polymerization by reversible addition–fragmentation chain transfer (RAFT).^48^ PNAM has seen use in the modification of membranes to reduce protein fouling^49^ and improve hemocompatiblity,^50^ conjugation with enzymes to increase their solubility,^51^ and as responsive assembled drug carriers.^52^

In this work, we aimed to compare nanotubular vectors composed of covalent or supramolecular backbones, to gain a greater understanding of the in vivo behavior of the cyclic peptide by assessing it against an equivalent low-MW linear polymer and BB structures. The non-degradable covalent backbone of the BBs means that the size can be accurately determined and will be maintained throughout in vivo environments, which therefore provides a useful comparison to the dynamic cyclic peptide conjugate system. Furthermore, the usage of PNAM-based systems were studied, to probe its potential as an alternative to PEG.

## Experimental (Materials and Methods)

### Materials

4-acryloylmorpholine (NAM, 97%) was obtained from Sigma-Aldrich and passed through a basic alumina column before use. *N*-Acrylic acid hydroxysuccinimide ester (NAS, >90%), 4,4′-azobis(4-cyanovaleric acid) (ACVA, >98%), acryloyl chloride (>97%), acetonitrile, trimethylamine, diisopropylethylamine (DIPEA), triisopropylsilane, dimethyl sulfoxide-*d*_6_ (99.9% D atom), and chloroform-*d* (99.8% D atom) were obtained from Sigma-Aldrich and used as received. CH_3_O-PEG-NH_2_ (MW 2000 Da, Rapp Polymere), Alexa-488 Cadaverine (Fisher), ^14^C-ethanolamine (50–60 μCi/mmol, American Radiolabeled Chemicals), 1,4-dioxane (anhydrous, Acros Organics), *N*,*N*-dimethylformamide (DMF, anhydrous, Acros Organics), *N*-methylmorpholine (NMM, Alfa Aesar), and piperidine (Alfa Aesar) were used as received. 4-(4,6-Dimethoxy-1,3,5-triazin-2-yl)-4-methylmorpholinium tetrafluoroborate (DMTMM·BF_4_), O-(1*H*-6-chlorobenzotriazole-1-yl)-1,1,3,3-tetramethyluronium hexafluorophosphate (HCTU), 2-chlorotrityl resin, Fmoc-d-Leu-OH, Fmoc-l-Lys(Boc)-OH, and Fmoc-l-Trp(Boc)-OH were purchased from Iris Biotech and used as received. Syntheses of the polymer brushes and the control polymer are reported in the Supporting Information.

### Instrumentation and Analysis

#### NMR Spectroscopy

^1^H and ^13^C NMR spectra were ran on either a Bruker DPX-300 or DPX-400 spectrometer using deuterated solvents (deuterated dimethyl sulfoxide, chloroform, or water).

#### SEC Analysis

Size exclusion chromatography (SEC) analysis was performed on two systems: DMF-SEC: Agilent 390-LC MDS instrument equipped with differential refractive index, viscometry, dual angle light scatter, and dual wavelength UV detectors. The system was equipped with 2 × PLgel Mixed D columns (300 × 7.5 mm) and a PLgel 5 μm guard column. The eluent is DMF with 5 mmol NH_4_BF_4_ additive. Samples were run at 1 mL min^–1^ at 50 °C. Poly(methyl methacrylate) standards (Agilent EasyVials) were used for calibration, MW ranging from 550 to 2.14 × 10^6^ g mol^–1^. Analyte samples were filtered through a nylon membrane with 0.22 μm pore size before injection. Experimental molar mass (*M*_n_,_SEC_) and dispersity (D̵) values of synthesized polymers were determined by conventional calibration using Agilent GPC/SEC software.

Dimethylacetamide (DMAC)-SEC was performed on a Shimadzu modular system comprising an SIL-20AD automatic injector, a RID-10A differential refractive-index detector, and a 50 × 7.8 mm guard column followed by three KF-805L columns (300 × 8 mm, bead size: 10 μm, pore size maximum: 5000 Å). *N*,*N*-dimethylacetamide (DMAc, 0.03% LiBr) was used as the eluent with a flow rate of 1 mL min^–1^ at 50 °C. Samples were filtered through 0.45 μm poly(tetrafluoroethylene) filters before injection. The SEC calibration was performed with polystyrene standards, ranging from 500 to 2 × 10^6^ g mol^–1^.

#### Atomic Force Microscopy

Atomic force microscopy (AFM) images were acquired in the AC mode on a Cypher S system (Asylum Research). The probes used were AC160TS from Olympus probes with a nominal resonant frequency of 300 kHz and a spring constant of approximately 40 N m^–1^ on a multimode AFM (Asylum Research). Images were acquired at a pixel resolution of 512 and a scan rate of 1 Hz. Samples were diluted to 1 mg mL^–1^ in water, and samples were prepared by drop casting the solution onto a freshly cleaved mica substrate and drying under stream of nitrogen. The data were analyzed with Gwyddion software.

#### Small-Angle Neutron Scattering

Small angle neutron scattering (SANS) experiments were performed on the SASN2D small-angle diffractometer at the ISIS Pulsed Neutron and Muon Source, Harwell, Didcot. A simultaneous Q-range of 0.0045–0.7 Å^–1^ was achieved, utilizing an incident wavelength range of 1.75–16.5 Å and employing an instrument set up of L1 = L2 = 4 m, with the 1 m^2^ detector offset vertically 60 mm and sideways 100 mm. The beam diameter was 8 mm. Models for fit are described in the Supporting Information.

### Synthesis of Linear Peptides

Synthesis of NH_2_-l-Lys(Boc)-d-Leu-l-Trp(Boc)-d-Leu-l-Lys(Boc)-d-Leu-l-Trp(Boc)-d-Leu-COOH was performed using a Prelude (Protein Technologies Inc.) automated solid phase peptide synthesizer, using a previously described procedure. 2-Chlorotrityl resin (0.36 g) was allowed to swell with DCM prior to loading by addition of a solution of Fmoc--leu-OH (1.01 g, 2.86 mmol) and DIPEA (0.4 M) in DMF (16 mL) and reacted for 2 h, drained, and then treated with DCM/MeOH/DIPEA (17:2:1, 10 mL) to ensure capping of unreacted resin sites. The drained resin was washed with DMF, and 20% Piperidine solution in DMF (15 mL) was added to deprotect the Fmoc groups, followed by further washing with DMF. Subsequent coupling steps were performed by addition of Fmoc-amino acid (2.86 mmol) with an HCTU (0.83 g, 0.20 mmol) and NMM (0.44 mL, 0.4 mmol) solution in DMF (10 mL), left to react for 2 h and then washed with DMF. Further deprotection and addition steps were repeated until the targeted octapeptide was synthesized. After the final Fmoc deprotection step, the peptide was cleaved from the resin by addition of hexafluoro-2-propanol (HFIP) (20%) in DCM (3 × 10 mL) and washed with DCM. The solution was concentrated under vacuum to yield an off-white solid. ESI MS +ve: calcd for [M + Na]^+^ 1503.89. *m*/*z* 1503.8 found.

### Cyclization and Boc Deprotection of Linear Peptide

The linear peptide (350 mg) was dissolved in DMF (100 mL), and DMTMM·BF_4_ (143 mg, 0.44 mmol) was added and stirred under nitrogen for 5 days at room temperature. The DMF was removed by concentration under reduced pressure, redissolved in DMF (10 mL), precipitated into ice-cold MeOH/H_2_O (1:1), and then dried in a vacuum oven overnight.

The solid was dissolved in a mixture of 95% trifluoroacetic acid (TFA, 5 mL), 2.5% triisopropylsilane, and 2.5% H_2_O, left to stir for 3 h, and the reaction mixture was transferred to a falcon tube and precipitated by addition of ice-cold diethyl ether. The supernatant was discarded, washed with additional diethyl ether, centrifuged (process repeated twice), and then transferred to a vacuum oven and left to dry overnight. The product was isolated as a pale orange powder (260 mg). ESI MS +ve: calcd for C_58_H_88_N_12_O_8_Na [M + Na]^+^ 1103.7. *m*/*z* 1103.6 found. See the Supporting Information for assigned ^1^H NMR.

### Chain Extension of a 2-Arm PNAM_37_ Cyclic Peptide Conjugate

The PNAM conjugate was prepared by amide coupling of the amine units of the deprotected cyclic peptide and an NHS ester of the end group of a PNAM_37_ polymer synthesized from a NHS functional RAFT agent. This compound (12.5 mg, 1.98 × 10^–3^ mmol) was then chain extended by addition of NAM (2 mg, 0.0149 mmol), NAS (2.4 mg, 0.0149 mmol), ACVA (0.1 mg, 4.72*10^–4^ mmol), and DMF (200 μL) in a 1 mL glass vial. The reaction mixture was degassed with nitrogen, placed in an oil bath heated to 70 °C, and stirred for 6 h, then precipitated twice into diethyl ether, redissolved in water, and freeze dried to yield a colorless powder.

### Radiolabeling by Conjugation of C14-Ethanolamine

A typical procedure is described: the PNAM-*co*-PNAS short brush (20 mg, 6.66 × 10^–3^ mmol NAS units), tetraethylammonium (TEA) (0.3 mg, 2.96 × 10^–3^ mmol), and ^14^C-ethanolamine (1.6 × 10^–3^ mmol, 0.4 mL solution in H_2_O/ethanol) were dissolved in DMF (0.5 mL) in a 3 mL screw cap vial and stirred at room temperature for 48 h, after which an additional unlabeled aliquot of ethanolamine (3 mg, 4.9 × 10^–2^ mmol) was added to the reaction mixture and stirred for a further 4 h. The reaction mixture was concentrated under a stream of nitrogen, redissolved in water, and immediately passed through a PD10 purification column collected into 1 mL fractions. These were analyzed by liquid scintillation counting to confirm the separation of polymer and free radiolabel; fractions 3–6 (polymeric species) were mixed and placed in a floatalyzer dialysis device with a 10 k MW cutoff and dialyzed against water for 3 days until no radioactivity in the bulk water was measurable. The solution was transferred to a vial, dried under a stream of nitrogen with heating at 50 °C to yield the radiolabeled conjugate as a white residue.

For the cyclic peptide labeling conjugate, the C14-ethanolamine was dried under a stream of nitrogen prior to use, the removal of water was found to improve the reaction yield presumably by reducing the hydrolysis rate of the NHS ester and was necessary for the acquirement of higher radioactivities, as required for the CP conjugate. CP-NAS (4 mg, 3.66 × 10^–3^ mmol), dried ^14^C-ethanolamine (1.6 × 10^–3^ mmol), and TEA (0.3 mg, 2.96 × 10^–3^ mmol) were mixed in a 3 mL screw cap vial. The same procedure as described above was carried out.

### Cells

MDA-MB-231, HEK-293, 4T1, 3T3 were obtained from the American Type Culture Collection (ATCC). MDA, HEK, and 4T1 were grown in Dulbecco’s modified Eagle’s medium (DMEM) supplemented with 10% v/v fetal calf serum. 3T3 was grown in DMEM supplemented with 20% v/v fetal calf serum. Cells were grown as adherent monolayers at 37 °C in a 5% CO_2_ humidified atmosphere and passaged at ∼80% confluence.

### Cell Proliferation Assay Protocol

Viability of cells in the presence of the synthesized compound was assessed by MTT assay. Cells were seeded in a 96-well plate (5000 cells per well) and allowed to grow for 24 h, followed by the addition of a solution of compound dissolved in cell culture media. The solutions were made up to give a concentration in the well media to a range of 0.001–1 mg mL^–1^. The cells were incubated for 72 h, after which the wells were treated with MTT compound [12 mM in phosphate-buffered saline (PBS), 10 μL] and incubated for 4 h. The culture media was then removed by aspiration, DMSO (50 μL) was added to each well, and the plate was incubated at 37 C for 10 min to fully dissolve the purple crystals formed upon the oxidation of the MTT reagent. The UV absorption at 540 nm was measured in a Flexstation 3 plate reader to determine the cell viability. The measurements were carried out by triplicate, and the results were normalized with the absorbance coming from the test performed on untreated cells.

### Cell Association by Flow Cytometry

Cells were seeded in a 24-well plate (100,000 cells per well) with 0.5 mL culture media and incubated for 24 h. A solution of Alexa-488 labeled compounds (75 μL, 1.5 mg mL^–1^) in culture media was dosed to the cells in triplicate under three experimental conditions: incubation at 37 °C for 3 or 24 h and incubation at 4 °C for 3 h. For the 4 °C experiment, the 24-well plate and sample solution were cooled on ice for 10 min prior to the dosage of the compound and kept on ice for the duration of incubation. After incubation, the culture media was then removed, and the cells were washed twice with cold PBS, treated with Trypsin, and incubated for 10 min to harvest them. A solution of bovine serum albumin (BSA, 10%, 0.3 mL) was added to each well, transferred to a 96-well plate, and centrifuged at 350 G for 5 min, after which the supernatant was discarded, and the cells were resuspended in 10% BSA solution. Samples were analyzed using a S100EXi flow cytometer (Stratedigm), equipped with 405, 488, 552, and 640 nm solid-state lasers. Forward and side scatter gates were used to exclude debris and dead cells using a viability dye (propidium iodide). The mean fluorescence intensity for a population of 10,000 cells was determined with Flowjo v8 for each experimental condition in triplicate.

### Spatial Coincidence with Lysotracker-Marked Lysosomes

Cells were seeded in an eight-well microscopy slide (20,000 cells per well) with 150 μL of culture media and incubated for 24 h at 37 °C. A solution of Alexa-488 labeled compounds (33 μL, 0.3 mg mL^–1^) in culture media was dosed to the cells and incubated for 8 h. Lysotracker Deep Red (ex/em: 647/668, 2 μL per well, 75 nM in DMEM) and Hoechst 33342 (ex/em: 361/497, 1 μL, 1 μg mL^–1^ in DMEM) were added 30 and 5 min before imaging the cells, respectively. After that, the cells were washed with media and imaged in a humidified incubation chamber with a regulated temperature of 37 °C using a Leica TCS SP8 Laser-scanning confocal microscope with a HCX PL APO 40× (NA 1.30) oil objective. Images were acquired at 1024 × 1024 with a pinhole set to 1 Airy units, capturing Lysotracker Deep Red (ex 633; em: 650–778 nm), AF488-brushes (ex 488; em 502–594 nm) and Hoechst 33342 (ex 405, 410–460 nm) fluorescence. Image acquisition settings were consistent for samples and controls. Images were processed with the FIJI distribution of Image J.

### In Vivo Pharmacokinetics Protocol

All animal experimental protocols were approved by the Monash Institute of Pharmaceutical Sciences Animal Ethics Committee, Monash University, Parkville, VIC, Australia. Male Sprague Dawley rats (250–350 g) were used. Animals were maintained on a 12 h light/dark cycle at all times and after transport were acclimatized for 7 days at the facility prior to in vivo studies.

A day prior to compound administration, each rat was anaesthetized under isoflurane (2–5% v/v) and cannulas (polyethylene tubing 0.96 × 0.58 mm, Paton Scientific, Victor Harbour, Australia) surgically inserted into the right jugular vein and carotid artery (to facilitate IV administration and blood collection, respectively), as previously described.^46^ The rats were transferred to individual metabolic cages (to permit separate collection of urine and feces) and allowed to recover overnight prior to dosing. Each animal was fasted up to 14 h prior to administration of the IV dose with water provided ad libitum. Prior to injection, blank blood samples (0.2 mL) were obtained from the carotid artery. The compounds were dissolved in PBS, and 0.5 mL was administered as a slow bolus (1 mL/min) at a dose of 1 μCi (2.8–9.1 mg kg^–1^) via the jugular cannula. The cannula was then flushed with 0.5 mL of heparinized saline after dose administration to ensure the complete infusion of the dose. Blood samples (0.2 mL) were taken prior to dose administration and at 1, 5, 10, 20, 30, 60, 120, 180, 240, 360, 480, and 1440 min after dose administration. Blood samples were placed immediately into tubes containing 10 IU of heparin and centrifuged for 5 min at 3500 g. Plasma (50 μL) was collected, transferred to a separate vial, and mixed with 4 mL of Ultima Gold scintillation cocktail prior to scintillation counting. Urine samples were collected at the 24 h timepoint, and a 50 μL of the sample was transferred to a vial, 4 mL of Ultima Gold added and counted using the scintillator. A blank urine sample prior to dosage was also collected and analyzed to account for background radiation.

The pharmacokinetic parameters were determined using non-compartmental analysis with Excel software using the PK Solver add-in (NCA IV Bolus model). Since the animals were dosed with varying mass but constant radioactivity (1 μCi), the concentrations in terms of μCi mL^–1^ of plasma were used and area under the curve (AUC) reported as μCi mL^–1^ min^–1^. AUC_0-*t*_, elimination half-life (*t*_1/2_), volume of distribution at steady state (*V*_d ss_), and clearance rate (Cl) were calculated with this approach. For the linear compound, the 1440 min timepoint was omitted from parameter fitting due to the very low radioactivity for this measurement.

### Biodistribution Protocol

After 24 h, the blood sample was collected, animals were humanely killed by the injection of a lethal dose of sodium pentobarbital via the jugular vein cannula and the liver, spleen, pancreas, kidneys, heart, lungs, and brain were harvested. The tissues were frozen and stored in polypropylene tubes prior to processing. The samples were homogenized with MilliQ water (5 mL) using a gentleMACS dissociator. Two triplicates of each organ sample (50–100 mg), one with and without addition of a known quantity of ^14^C-ethanolamine spike, were mixed with solvable (2 mL, PerkinElmer) and the samples heated at 60 °C overnight. After cooling to room temperature, hydrogen peroxide (200 μL, 30% w/v) was added to each vial, followed by addition of Ultima Gold scintillation cocktail (10 mL), vortexed, and stored at 4 °C for 3 days prior to counting. A sample of blank organs was also processed and analyzed in the same manner to provide a background correction. To account for the loss of activity as a result of the processing, an efficiency was then calculated to better determine the true dosage per organ
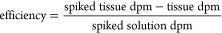
where “spiked tissue dpm” is the average measured degradation per minute of the spiked sample, “tissue dpm” is the average measure of the unspiked sample, and “spiked solution dpm” is the known amount of the radiolabel spike added. The efficiency was then used to correct for the true ^14^C content by
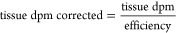


This value was then used to determine the total dpm of the organ by taking into account the total organ mass prior to processing of which 50–100 mg was analyzed in each sample. The results are reported as either the % dosage per organ or % dosage per gram tissue.

## Results and Discussion

### Design and Synthesis

**Figure 1:**
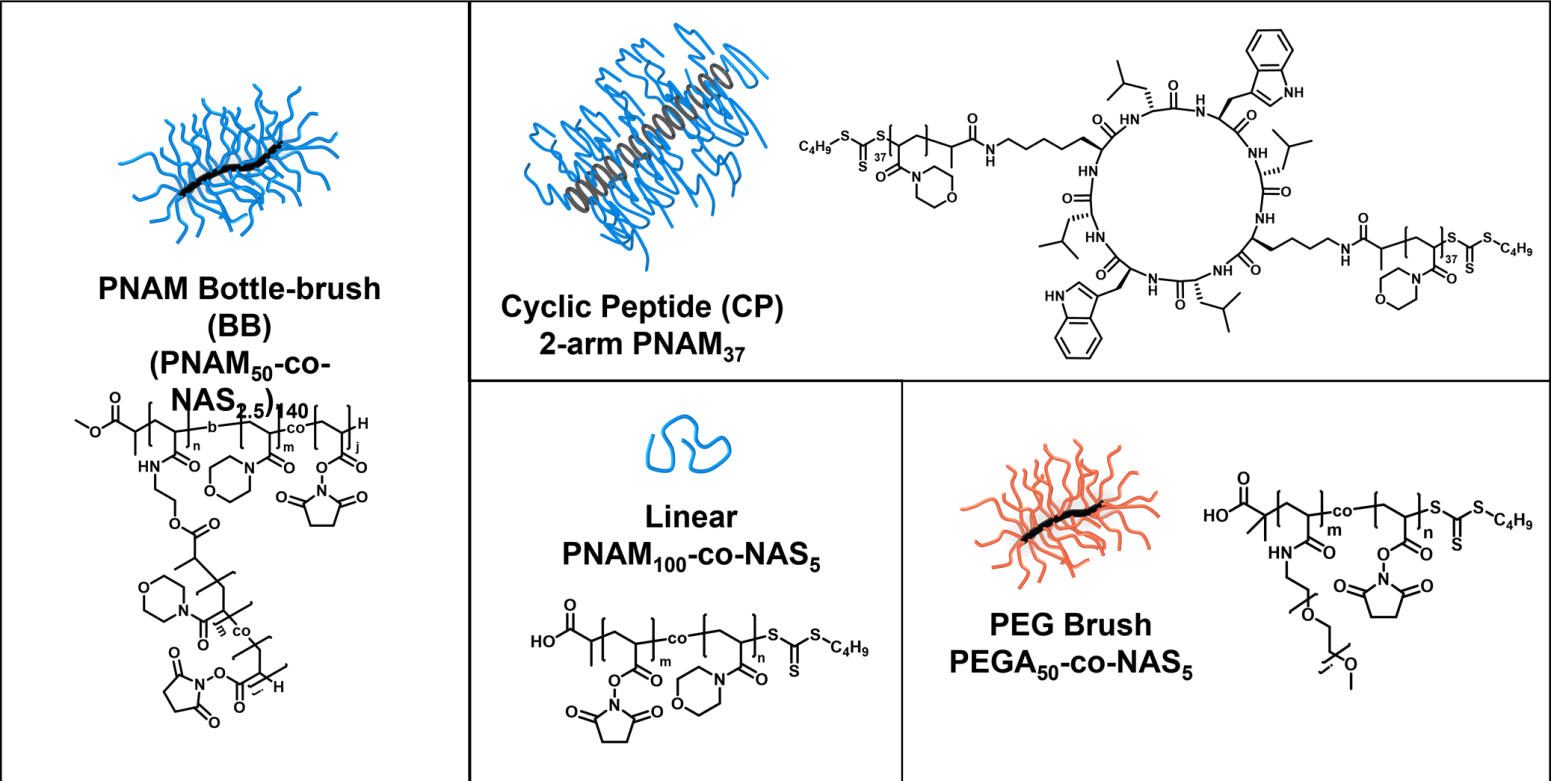
Scheme and chemical structures of the four compounds studied in this work.

For the assessment of the biomedical potential of BBs and nanotube systems, the cytotoxicity, pharmacokinetic, and biodistribution behavior of the unmodified materials must first be studied. Once established, the most suitable candidates for loading with active drug molecules could be selected. Therefore, four compounds were designed for the study—a supramolecular PNAM-cyclic peptide nanotube, a PNAM-composed BB, a covalent PEG-based BB to assess PNAM against a known standard, and finally a short low MW linear PNAM to act a as control (Figure 1).

**Figure 2:**
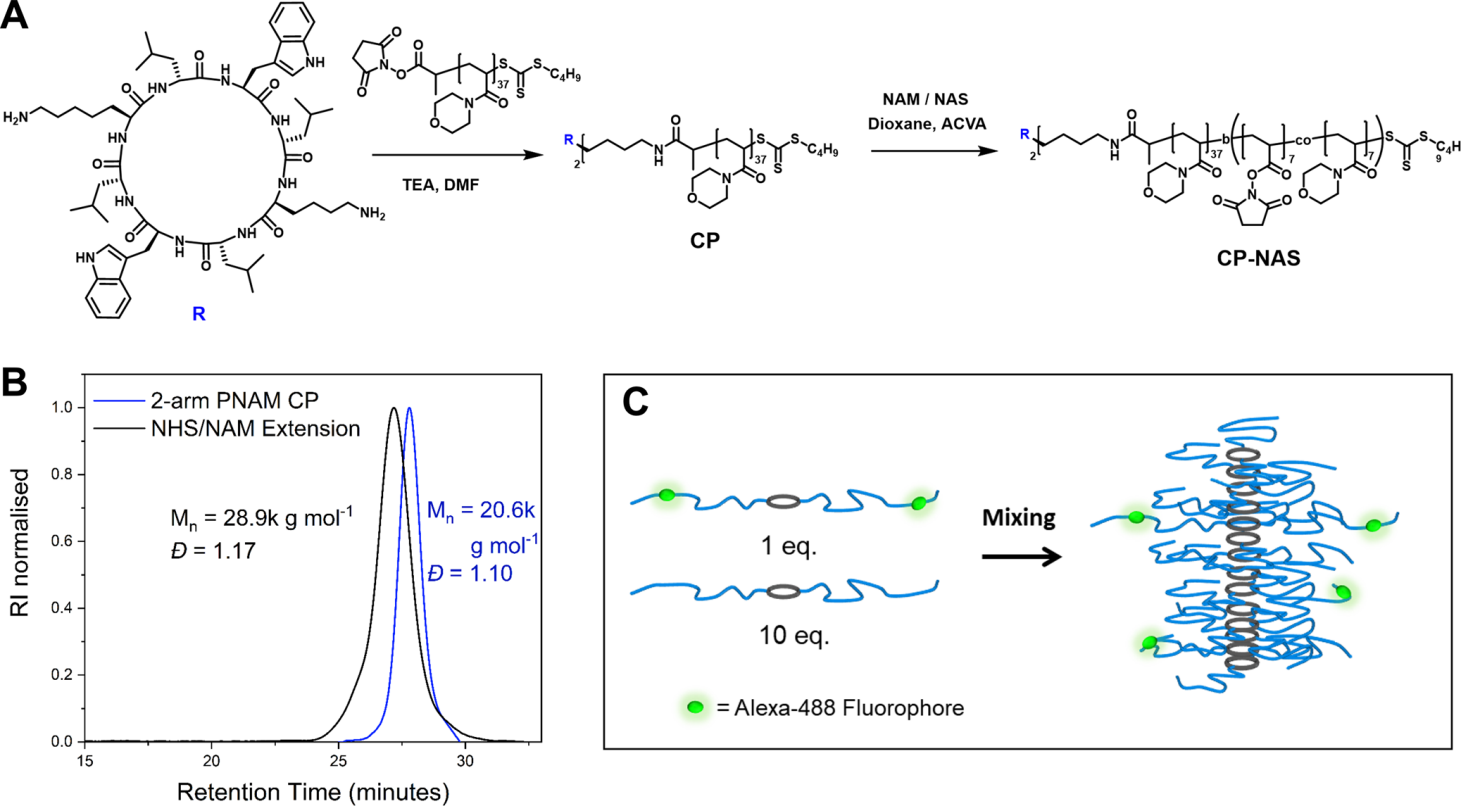
(A) Synthetic approach of the CP conjugate, and chain extension with NAS units to enable the subsequent labeling. (B) SEC chromatograms in the DMAc eluent system, showing clear chain extension of the conjugate with the NAS containing block. (C) Schematic for the mixing approach of labeled CP with the starting material.

PNAM-conjugated cyclic peptides were synthesized from a RAFT grafting to approach with two polymeric “arms” per peptide core, by conjugation of an end-functional NHS ester RAFT agent to the amino side groups of the cyclic peptide. The PNAM conjugates were found to readily self-assemble into nanotubes (36 nm in length), with a significantly higher aggregation number than previously studied PHPMA conjugates,^37^ and were therefore promising as nanotubular delivery candidates. Assembled cyclic peptide nanotubes possess a 0.47 nm distance between the peptide repeating units, whereas for vinyl backbone derived BBs, where a side chain is attached on every backbone unit, there is a 0.25 nm repeat unit distance between each side chain. Therefore, the grafting density of a 2-arm polymer cyclic peptide conjugate should be approximately the same to that of a singly grafted BB. Previous research identified that the RAFT grafting from the approach can yield BBs of high grafting densities (>90%); therefore, the actual repeat unit distance may be slightly higher than 0.25 nm but within a comparable range.^53^ To enable the labeling of the constructs with either tracing moieties, NAS monomer units were incorporated, which are known to copolymerize with NAM in an almost perfectly statistical manner (Figure S1).^54^ In the case of the cyclic peptide, after synthesis of the PNAM CP conjugate, the side arms were further chain extended by RAFT polymerization with a PNAM_7_-*co*-PNAS_7_ block (Figure 2).

**Figure 3:**
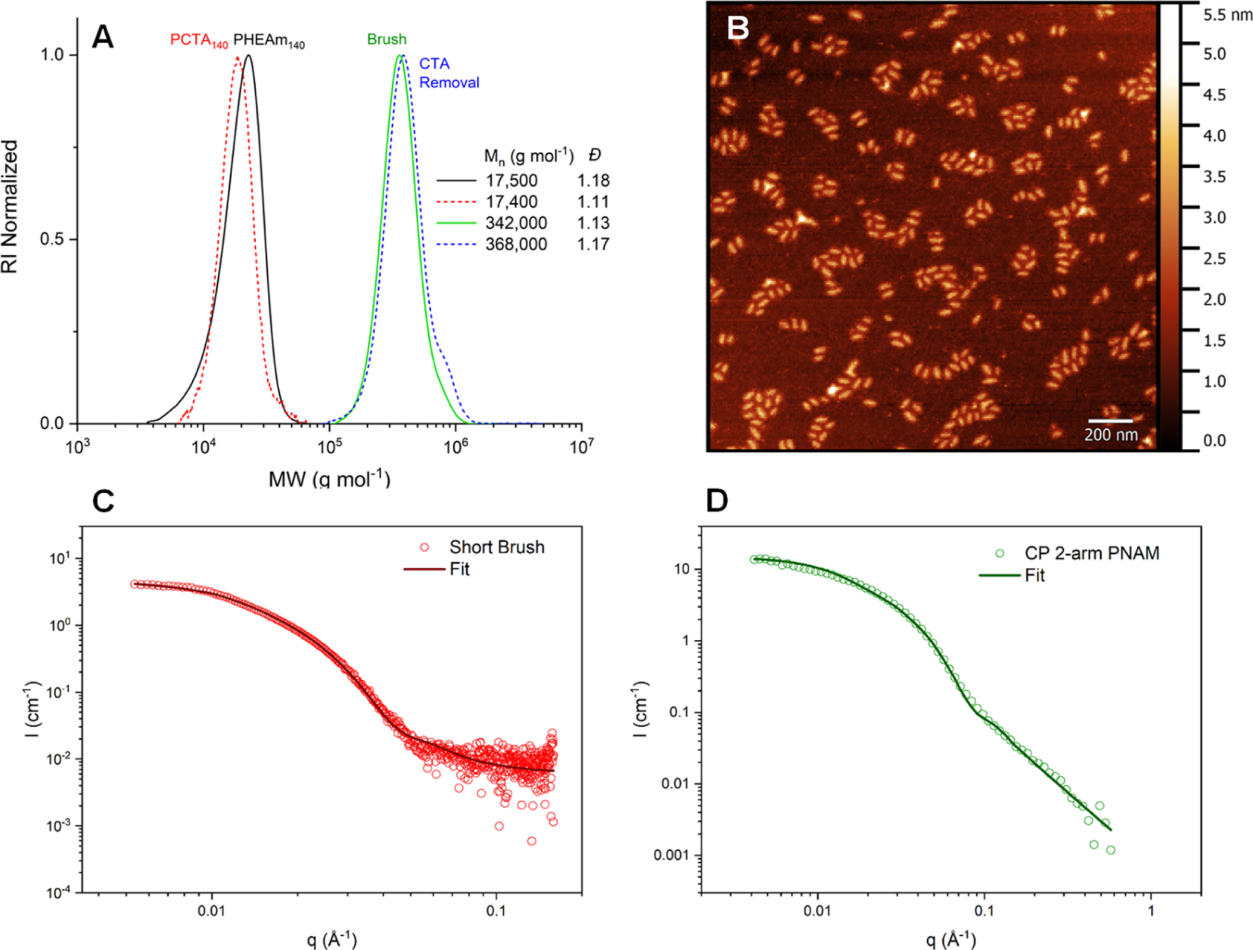
(A) SEC MW distributions using the DMF eluent system of each synthetic step for the PNAM BB, (B) AFM image prepared by drop casting a dilute aqueous solution of BB onto the mica substrate. (C) SAXS scattering profile of the BB (red) with the fitted models shown as a line. (D) SANS scattering profile of the CP 2-arm PAN conjugate and fit.

The RAFT R group *grafting from* the approach was used to synthesize the PNAM **BB** with a targeted backbone length of DP140 and a side chain length of DP50.^55^ In the grafting form step, a comonomer mixture of NAM/NAS (95:5 M ratio) was used, quenching the polymerization at ∼25% monomer conversion to mitigate brush–brush coupling terminations. SEC analysis reveals a single population with no low-MW linear polymer and narrow dispersity (*D̵* = 1.13) (Figure 3A). The aqueous solubility of the BB was initially poor; however, after end group removal of the butyltrithiocarbonate units by radical reduction with ACVA/LPO, the materials were fully soluble (Figure S3).

AFM analysis showed a well-defined backbone length of average 40 nm for the **BB** material, matching closely to the expected size for a fully extended DP140 backbone. The covalently composed **BB** therefore provides a controlled comparison of size to the dynamic **CP**. Small-angle scattering profiles of **CP** and **BB** could be fitted with a cylinder + Gaussian chain model (Figure 3C,D). The fitted length of the CP cylinder (36 nm) was very similar to the SB compound (41 nm) and thus should provide an effective comparison, while the magnitude of cylindrical radius was comparable (**CP**—4.1 nm and BB—8.1 nm).

A linear polymer of PNAM_100_-*co*-PNAS_5_ was selected as a control for the cyclic peptide conjugate, with a similar MW but lacking the self-assembly enabling peptide core. Difference in behavior between this compound and the CP conjugate can therefore primarily be attributed to stacking interactions from the core unit, leading to an increased hydrodynamic volume. Finally, a PEG BB was synthesized by grafting through of a 2000 g mol^–1^ PEG acrylamide macromonomer—44 PEG repeat units in each side chain is similar to the 50 PNAM repeat units targeted for the other materials. Unfortunately, targeting higher MWs for the macromonomer polymerization (>DP100) led to substantial broadening of dispersity (>1.3) (Figure S9), and therefore, a lower MW brush (*M*_n theo_ = 90,000 g mol^–1^) consisting of a DP50 backbone was studied (Table 1).

**Table 1:** Chemical Structure and Abbreviations of the Synthesized Compounds in This Study^a^

compound	structure	*M*_n theo_ (g mol^–1^)	*M*_n SEC_ (g mol^–1^)	*D̵*	activity (μCi mg^–1^)
**BB**	(PNAM_50_-*co*-NAS_2.5_)_140_	987,000	368,000	1.17	1.21
linear	PNAM_100_-*co*-NAS_5_	15,100	16,400	1.13	0.69
PEG	PEGAm_45_-*co*-NAS_5_	90,000	64,600	1.32	0.36
CP	CP-(PNAM_37_)_2_	12,000	20,600	1.10	
CP-NAS	CP((PNAM_37_)-*b*-(PNAM_7.5_-*co*-NAS_7.5_))_2_	16,400	28,900	1.17	6.59

a *M*_n SEC_ and *Đ* were determined using a DMF eluent system. Activities of the labeled materials were determined after reaction of the NAS units with C14-ethanolamine by liquid scintillation counting.

### Fluorescence and Radioactive Tag Labeling

**Figure 4:**
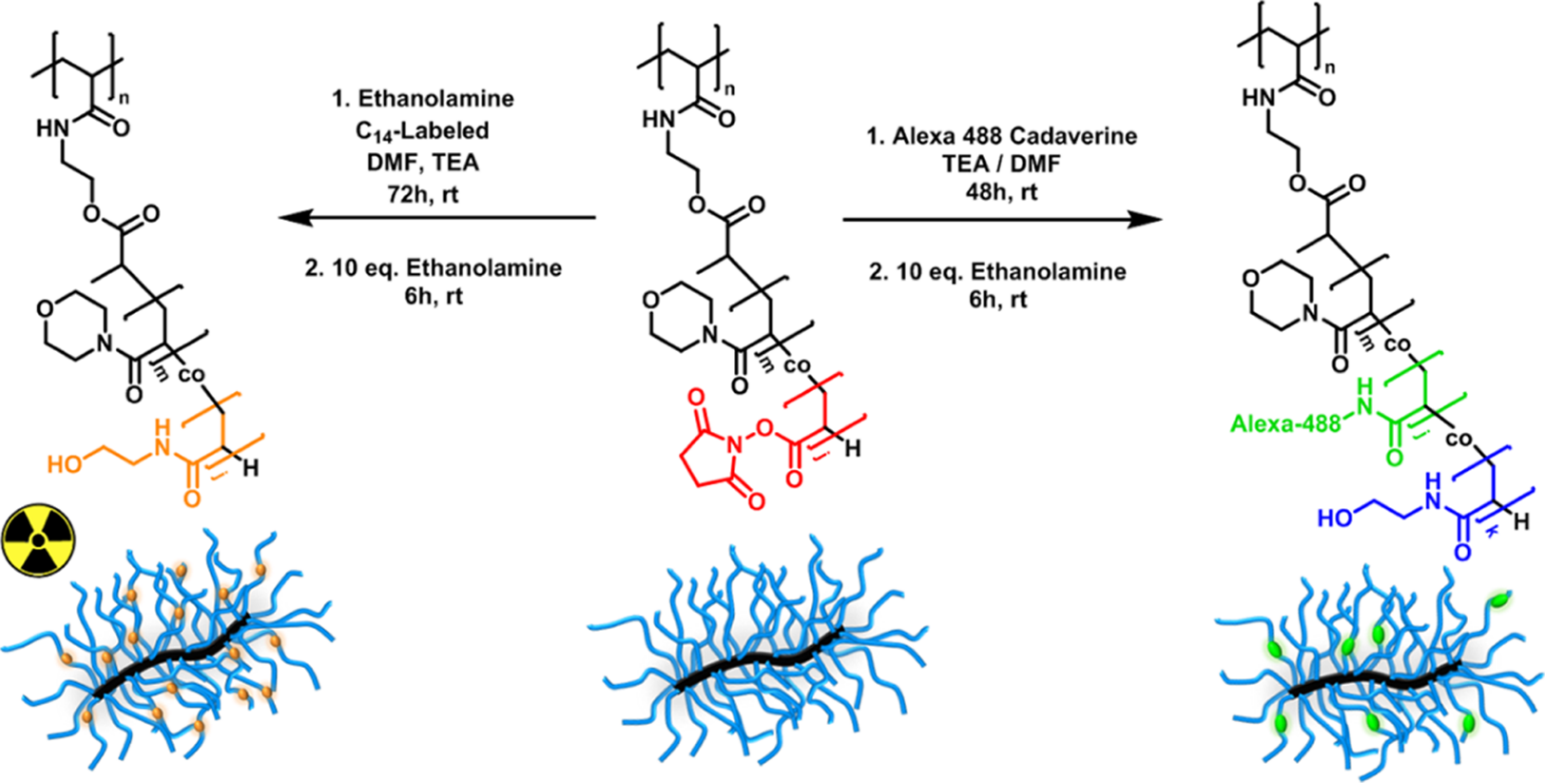
Synthetic scheme for the labeling of the PNAM brush with either a radioactive or fluorescent tag. The same reaction conditions were used for conjugation of the other four polymer materials.

To aid in the detection of the polymer constructs, either ^14^C radiolabeled ethanolamine or Alexa-488 fluorescent dye (excitation 488 nm, emission 502–594 nm) was conjugated to the polymer constructs by amide coupling of the amine functional label and NHS ester of the NAS monomer units (Figure 4). The high stability of the amide bond linkage should prevent label cleaving during experiments. The chain extended cyclic peptide conjugate (**CP-NAS**) was functionalized with a labeling group and then mixed in a 1:10 M ratio with the unlabeled **CP** starting material, with the dynamic nature of the cyclic peptide stacking interactions ensuring random co-assembly with the unlabeled conjugates (Figure 2C). A study on the dynamic co-assembly of cyclic peptides through FRET pair interactions has confirmed this behavior.^56^ The rationale of this approach was to mitigate the effects of labeling on the size of **CP** self-assembly—by decreasing the ratio of labeled conjugates and also by functionalizing at the exterior of the side chains, effects on the core hydrogen bonding should be avoided. The loading rate of label onto **CP-NAS** was targeted to be approximately 10 times higher than that of the BB/PEG/linear compounds, such that after mixing with the unlabeled **CP**, the resulting formulation will contain a comparable concentration of the labeling moiety.

Synthesis of fluorescently labeled compounds was performed with 0.015 equiv Alexa-488 with respect to NAS units (0.07 equiv for **CP**), after which a large excess of ethanolamine (unlabeled) was added to ensure full consumption of NHS ester units and prevent potential side reactions occurring during the biological experiments, while also ensuring the polymer remains fully hydrophilic with no residual hydrophobic NAS units. The low quantity of Alexa-488 moieties (>0.1% with respect to NAM monomer units) introduced onto the polymers reduces any potential change in the properties of the PNAM homopolymer endowed by the dye molecules. High-performance liquid chromatography (HPLC) of the Alexa-488 polymer conjugates confirmed fluorescent activity at the desired wavelengths and almost full removal of free dye molecules after dialysis purification (Figure S14).

**Figure 5:**
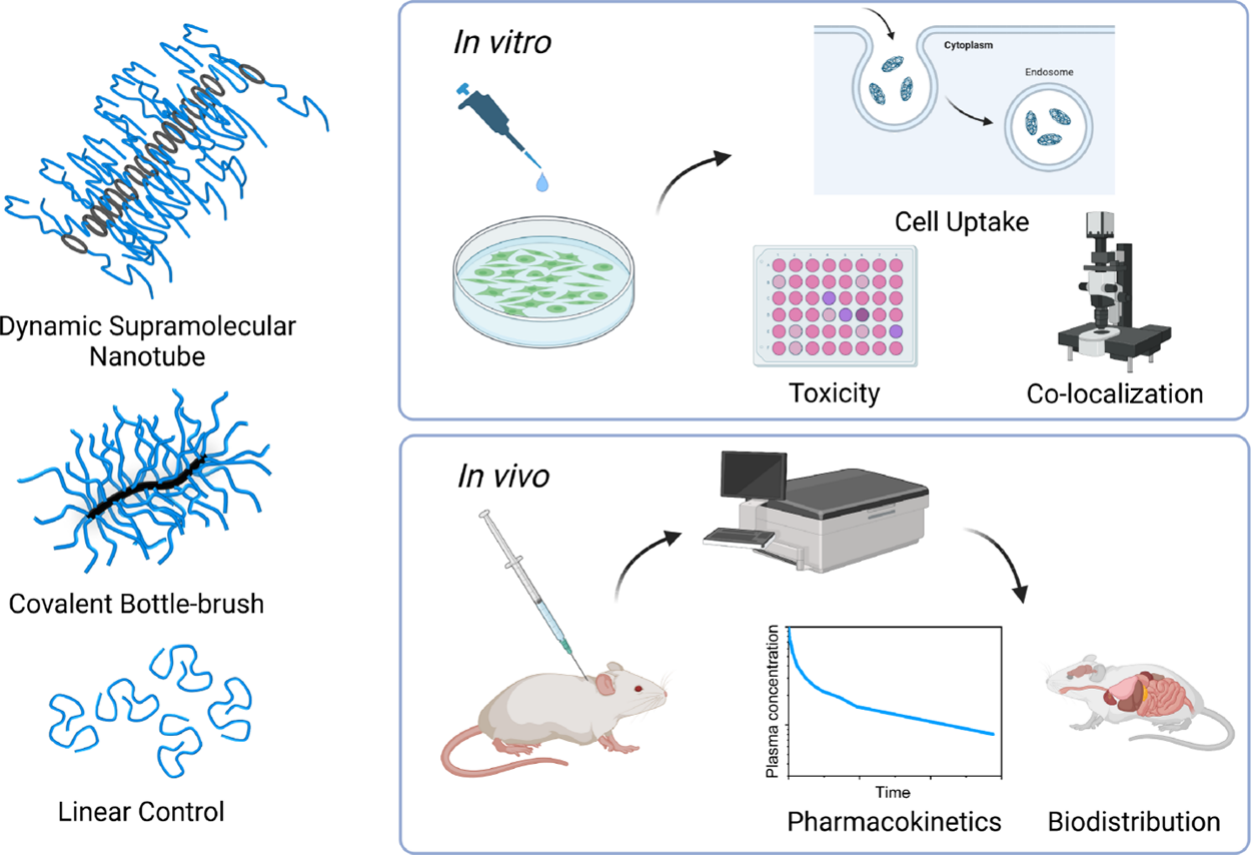
Summary of biological assays performed to compare the behavior of the cyclic peptide supramolecular nanotubes with the covalently constructed BB material. Created with BioRender.com.

Radiolabeling was performed targeting an activity of 1 μCi mg^–1^ by addition of 0.2 equivalents ^14^C-ethanolamine with respect to NAS units, excluding **CP** (see below). Purification by SEC (Sephadex PD10 column) and subsequent dialysis were carried out to ensure the complete removal of the free radiolabel, and indeed further SEC analysis (Sephadex G25 column) showed high sample purity (Figure S16). Some variations in the radioactivities were obtained (Table 1), with the linear and PEG compounds being significantly lower than the targeted ratio. The low conjugation efficiency found for the PEG compound is presumably due to the lower NAS content by weight (10 mol %, 0.9 wt %) in comparison to the PNAM copolymers (5 mol % and 3.9 wt %) since the activities are reported in units of μCi mg^–1^. An excess of NHS ester with respect to ethanolamine was used in all access, which should favor high conjugation of the radiolabel, suggesting that the sub-quantitative conjugation efficiency may arise from side reactions such as NHS ester hydrolysis. The mixing strategy for **CP** required higher activities of the labeled conjugate, and therefore, an increased value was targeted, with the purified conjugated possessing an activity of 6.59 μCi mg^–1^. The successfully labeled compounds were then taken forward to in vitro/in vivo study (Table 1, Figure 5).

### Cell Viability

The cytotoxicity of the polymer samples was assessed in vitro by MTT cell growth inhibition assay for 72 h on 3T3 fibroblast, MDA-MB-231 breast cancer, HEK-293, and 4T1 mouse breast cancer cell lines. PEG and PNAM are considered to be biocompatible polymers with low toxicities and as such similar results were expected for the compounds studied here.^46^ Prior to cell testing, all unlabeled compounds were reacted with standard ethanolamine to convert the NAS monomeric units into poly(hydroxyethylacrylamide) units, which has also been identified as a suitable biocompatible polymer.^57^ The products are thus chemically identical to the ^14^C-radiolabeled counterparts used in vivo. A minor degree of toxicity was observed at the higher dosages (1 mg mL^–1^) across all compounds (Figure 5), most clearly for the 4T1 cell line with cell viabilities dropping to ∼75%. For the further flow cytometry and confocal microscopy experiments, cells were dosed to a concentration of 0.3 mg mL^–1^ sample to minimize the detrimental toxicity.

### Cell Association

**Figure 6:**
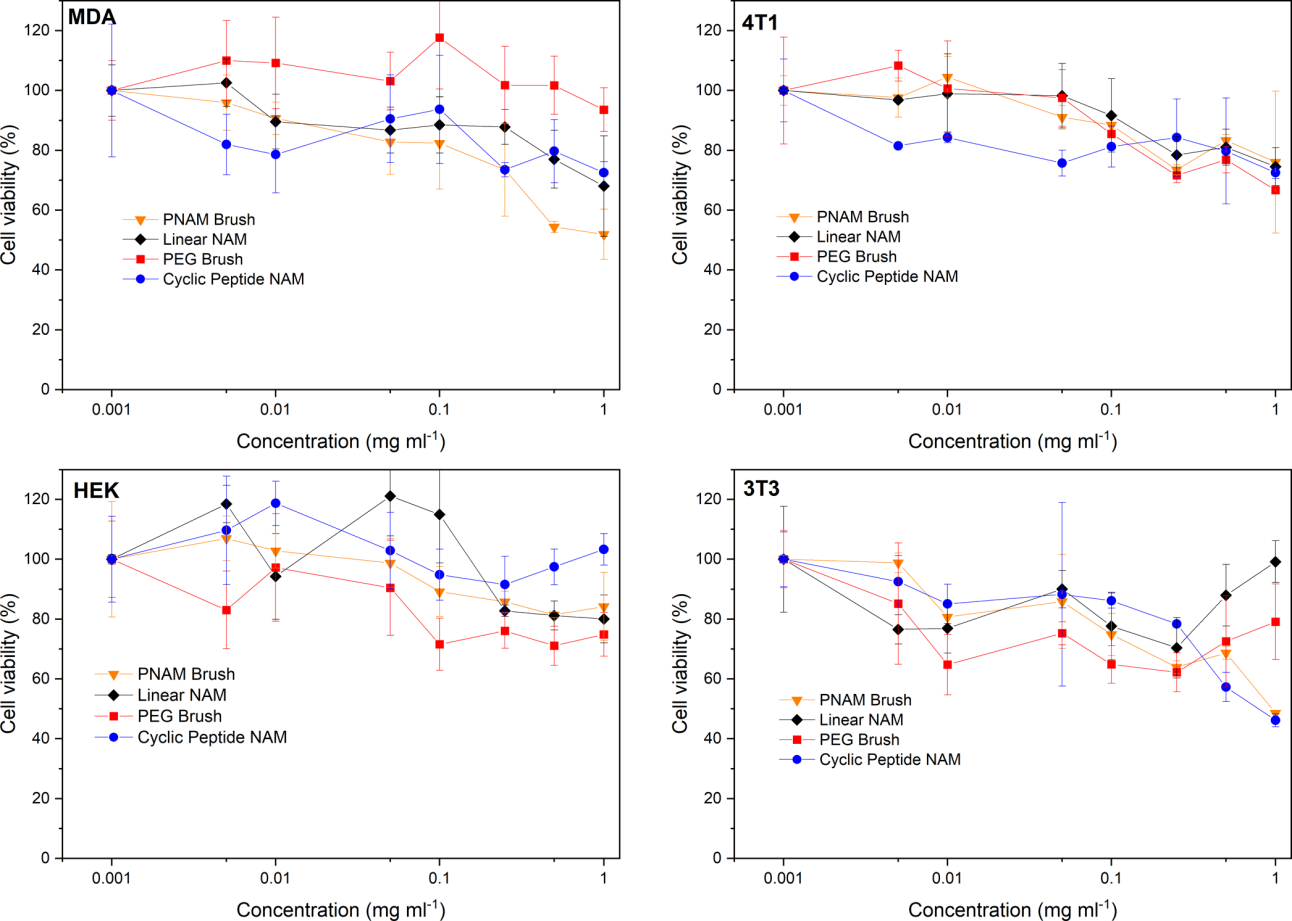
MTT assay data for four cell lines for the four compounds studied, 72 h timepoint, *n* = 3.

The Alexa-488 labeled polymers were used to determine cellular association by flow cytometry on two cell lines (MDA and 3T3). Cells were incubated with samples for 3 h at either 4 or 37 °C or for 24 h at 37 °C. All compounds showed the trend of significantly increased association going from 3 to 24 h, indicating the accumulation of the compound either adsorbed in the membrane or inside a cell compartment, typical for nanoparticle species. Low cellular fluorescence for the 3 h, 4 °C experimental condition implies that the uptake proceeds through predominately energy-dependent endocytosis pathways (Figure 6) rather than passive mechanisms such as membrane diffusion.

Poly(hydroxypropylmethacrylamide) cyclic peptide conjugates have previously been shown to display increased cell uptakes over an equivalent linear polymer.^37^ In the present case with PNAM, however, the **CP** and linear show comparable values after 3 and 24 h for both cell lines. Both the CP and linear samples display increased association over the **BB**, most notably in the 3T3 cell line.

A similar chemical composition of **BB** and linear compounds facilitates the determination of the polymeric structural effect upon cell association. Despite reports of nanoparticles in the 30–50 nm regime possessing high cell uptake compared to smaller/larger particles,^58^ in this case, the linear polymer (<10 nm *R*_h_) exhibits the highest association. As a result of the directional hydrogen bonding of the peptide core, the CP nanotubes are expected to be more rigid than their BB counterparts, where the polymer backbone will remain somewhat flexible. Recent studies have identified the importance of nanoparticle stiffness on cell uptake behavior, which could explain the significant difference between the BB and CP results despite their otherwise comparable morphology.^25,59,60^ Additionally, the dynamic nature of the CP assembly means that a proportion of low MW unimers is present in solution, of similar size to the linear sample, which is shown to undergo high association. As such, it is not possible to conclude whether the effect of supramolecular stacking into nanotubes has an impact on the association behavior of the PNAM conjugate. To fully understand this system, further studies would be required to elucidate the effects of the polymer composition (PEG/PNAM), the MW, and the predominant endocytosis mechanism.^61^

### Co-localization with the Lysosomal Tracker

**Figure 7:**
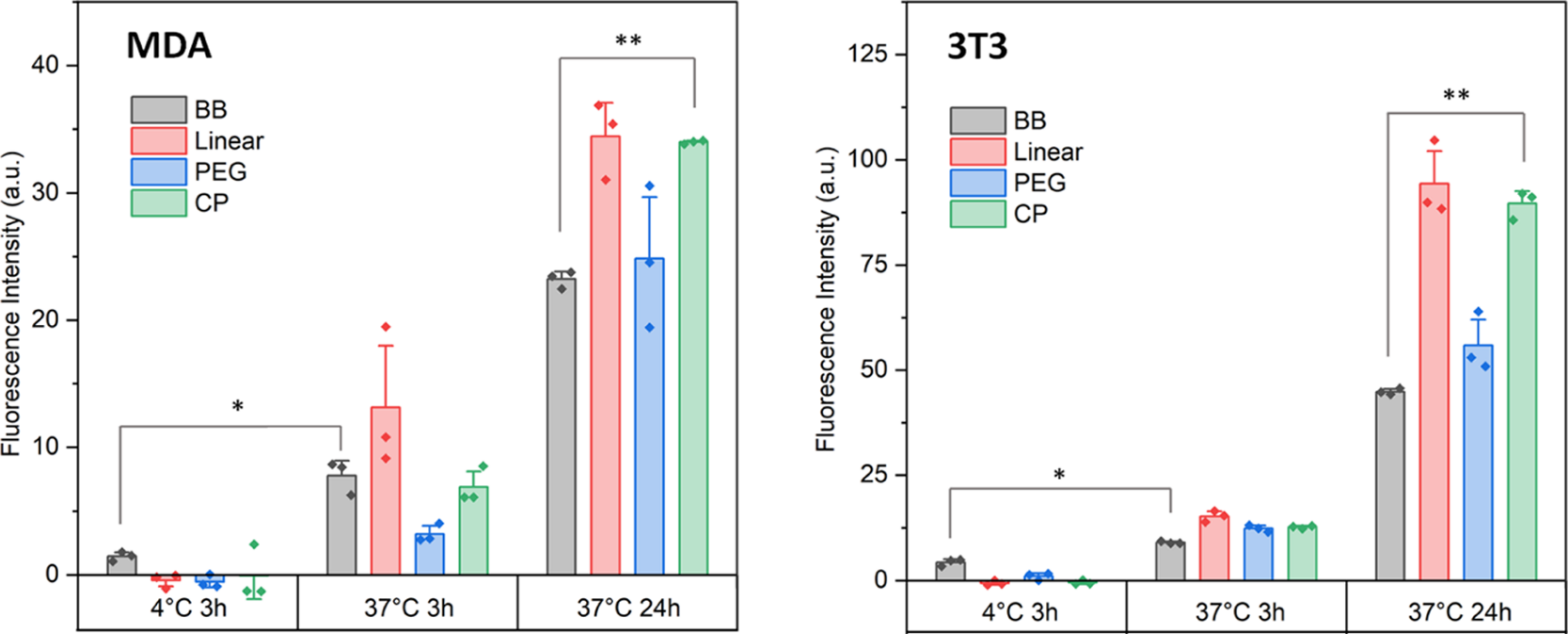
Flow cytometry data of cell lines treated with solutions of fluorescently labeled compounds at differing temperatures and timepoints for left—MDA and right—3T3 cell lines. Experiments were performed in triplicate for each set of conditions. Error bars indicate standard error. Statistical analysis with the Student *t* test: *p < 0.05 and **p < 0.005.

To gain more information on the cell association behavior, confocal microscopy was performed. Cell lines (MDA and 3T3) were incubated for 24 h with the fluorescently labeled conjugates and then co-treated with Lysotracker to identify any co-localization of the vectors with the lysosomal compartment. The presence of alexa-488 fluorescence (at the green channel, 488 nm) close to the cell nucleus (blue channel) confirms the uptake of compound inside the cell, rather than purely through the interaction with the membrane (Figure 7A). Treatment with Lysotracker Red enables the assessment of compound co-localization, and in the merged red and green channels, evidence of overlaying signal is observed by the presence of the yellow-colored regions. Time-lapse images were consistent with this observation (Figure S17). For all compounds across both cell lines, the uptake seems to occur primarily through the lysosomal compartments, consistent with the energy-dependent endocytic pathway as the most probable mechanism for cell association.

### Determination of Pharmacokinetics in Rats

The ^14^C-radiolabeled compounds were injected intravenously into male Sprague-Dawley rats at a nominal dose of radioactivity (1 μCi, varying 0.82–2.78 mg dose of the sample) and blood samples taken over the course of 24 h to monitor the plasma concentration time profiles. Non-compartmental pharmacokinetic parameters were calculated, and the values are listed in Table 2.

**Table 2:** Pharmacokinetic Parameters Determined by the Non-compartment Model Showing the Average and Standard Deviation across *n* = 3 In Vivo Plasma Concentration Experiments

	BB	linear	PEG brush	CP
*t*_1/2_ (h)	55.6 ± 27.6	2.7 ± 0.9	28.6 ± 9.4	14.6 ± 2.9
AUC (μCi mL^–1^ min)	50.75 ± 2.9	3.2 ± 0.4	63.5 ± 5.0	20.5 ± 0.8
Vss (mL)	24.3 ± 2.3	54.6 ± 27.9	16.8 ± 2.7	38.2 ± 3.5
Cl (mL h^–1^)	0.342 ± 0.102	16.9 ± 0.91	0.426 ± 0.065	2.06 ± 0.248
urine (% dose)	0.62 ± 0.10	50.8 ± 18.3	2.52 ± 0.14	28.5 ± 19.3

**Figure 8:**
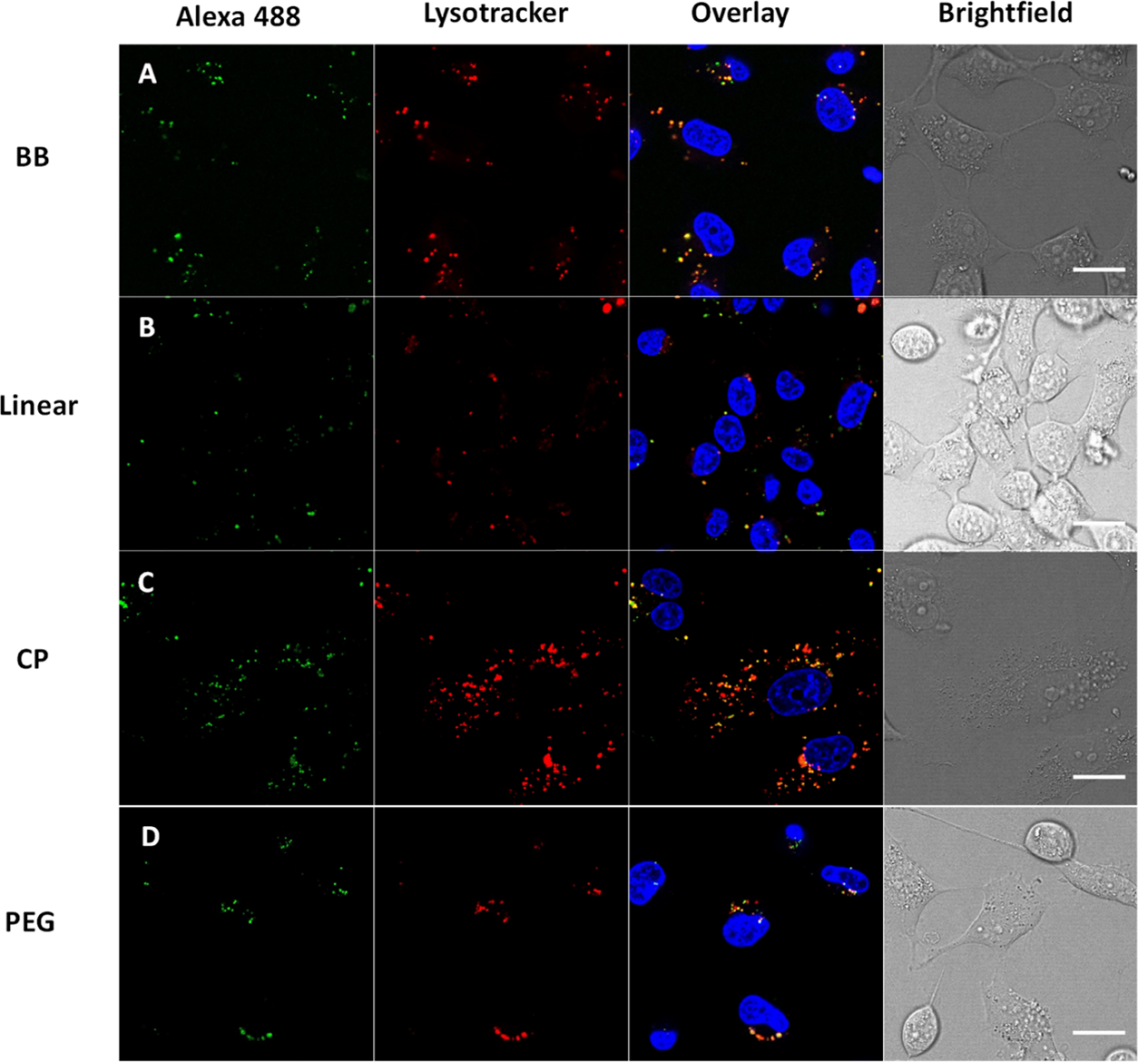
Confocal microscopy images of MDA cell cultures incubated with the labeled compounds for 24 h at 37 °C prior to imaging and with Lysotracker red/Hoechst blue stains shortly before. Channels show the Alexa-488 labeled compounds at 490/530 nm excitation/emission, Lysotracker at 647/668 nm, and Hoechst blue at 361/497 nm.

**Figure 9:**
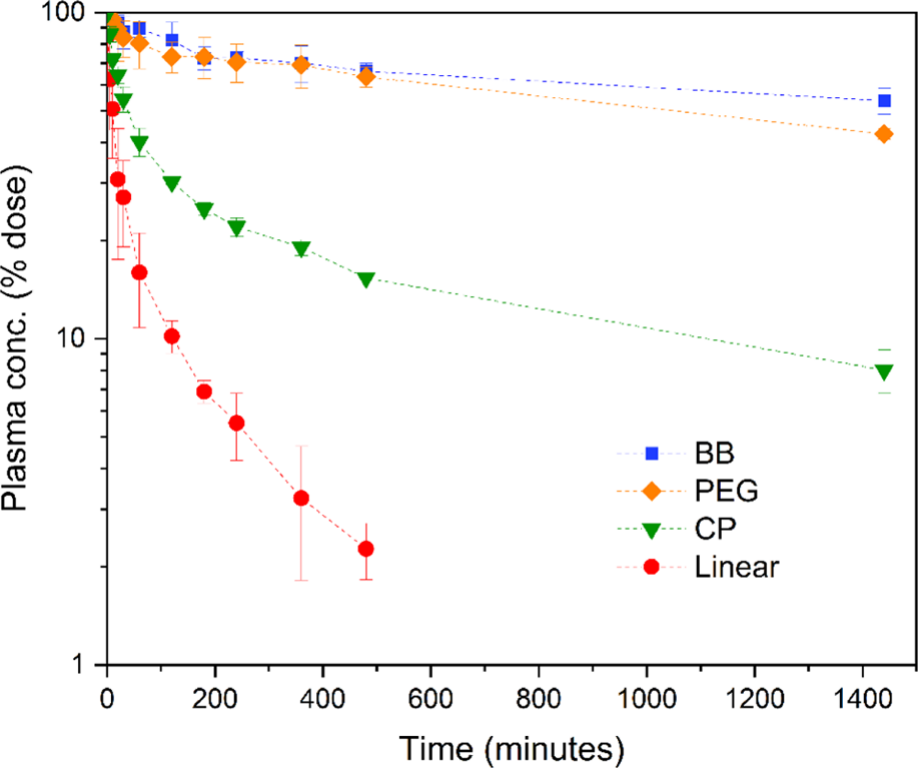
Plasma concentration profiles as a percent of the initial dose against time for the radiolabeled compounds. Sample solutions in PBS were injected into Sprague Dawley rats (concentration 2.8–9.1 mg kg^–1^, *n* = 3), blood samples withdrawn at timepoints and radioactivity assessed by scintillator counting.

Comparing percent dose normalized plasma concentrations (Figure 8), the linear polymer was cleared rapidly relative to the other polymers, whereas the BB polymers displayed higher exposure over time. This was reflected by their elimination half-life (2.7 h vs >35 h for linear and **BB**, respectively) and clearance values (Table 2). Interestingly, **CP** possessed intermediate values for both half-life time (14.6 h) and rate of clearance. The species with the higher clearance (linear and **CP**) also showed higher % dose recovered in urine (50.8 and 28.5% respectively), consistent with higher renal clearance. The significantly increased half-life of the **CP** conjugate compared to the linear is hypothesized to be caused by the stacking interactions of the cyclic peptide core moiety occurring in vivo, leading to a higher MW species that cannot be renally cleared. The higher circulation time of the **BB** (40 nm length), however, suggests that the **CP** conjugate either forms smaller nanotubes in vivo than the SANS determined length (36 nm) or is gradually disassembled as a result of the dynamic nature of the supramolecular interactions. The unimeric **CP** conjugate species is of comparable MW to the linear polymer and is thus expected to be similarly rapidly cleared. The nanotube disassembly may be partially attributed to the location within the vascular environment, where competitive hydrogen bonding interactions may occur with blood components in addition to shear flow forces (Figure 9).

Clearance values were very similar for the brush polymers, suggesting that the most important factor affecting circulation times is exceeding the renal excretion limit cutoff (∼5.5 nm *R*_h_, ∼50 kg mol^–1^ for a hydrophilic polymer)^62^ since both brushes exceed this value (1 M/90k g mol^–1^ for **BB**/**PEG**, respectively). The MW cutoff is approximate, however, and the reference above refers to a linear polymer—the highly branched systems studied here may deviate from this, as has been previously found for dendrimers where a MW cutoff of ∼40 kg mol^–1^ was observed.^63^ The slightly reduced plasma residence time of the PEG brush can be explained by the lower MW of this compound, which shows a higher % dose excreted into urine (2.5 vs 0.6% for SB). The MW distribution of the PEG brush (*M*_n theo_ = 90 000 g mol^–1^ and *M*_n SEC_ = 64 400 g mol^–1^, *Đ* = 1.32) indicates that a small proportion of species will fall below the excretion limit and thus may be cleared at a faster rate.

The apparent volume of distribution at steady state (*V*_d(SS)_) relates the mass of drug in the body to the concentration measured in blood under steady-state conditions. In the case of the high MW BB species, the *V*_d(SS)_ is expected to be close to the plasma/blood volume of the rat (∼12–24 mL) since the large hydrophilic brush is expected to distribute into the blood stream but not to extravasate beyond the vascular compartment. Values of *V*_d_ larger than the maximum blood volume suggest some degree of extravasation. For the **BB** and **PEG***V*_d(SS)_, values similar to blood volume were also observed (16.8–24.3 mL). A higher value was apparent for the linear polymer (89.5 mL), suggesting greater distribution for this lower MW construct. The **CP** system exhibited an intermediate *V*_d(SS)_ value (39.7 mL), higher than expected should the assembled system remain fully intact in vivo. The *V*_d(SS)_ data are therefore also consistent with the explanation that **CP** does form nano-assemblies, but that it also disassembles in vivo, allowing for both distribution and clearance via the renal excretion pathway (as demonstrated by high urine recovery of both the linear and **CP** compounds). The improved circulation time of **CP** over the linear polymer, along with moderate clearance rates may be promising for drug delivery applications, although the half-life is still significantly shorter than higher molar mass BBs. The comparable exposure times observed with the PNAM BB suggests that the material may have potential as an alternative to PEG in biomedical applications, although further detailed studies are required to assess effects such as the ABC phenomenon.

### Biodistribution

**Figure 10:**
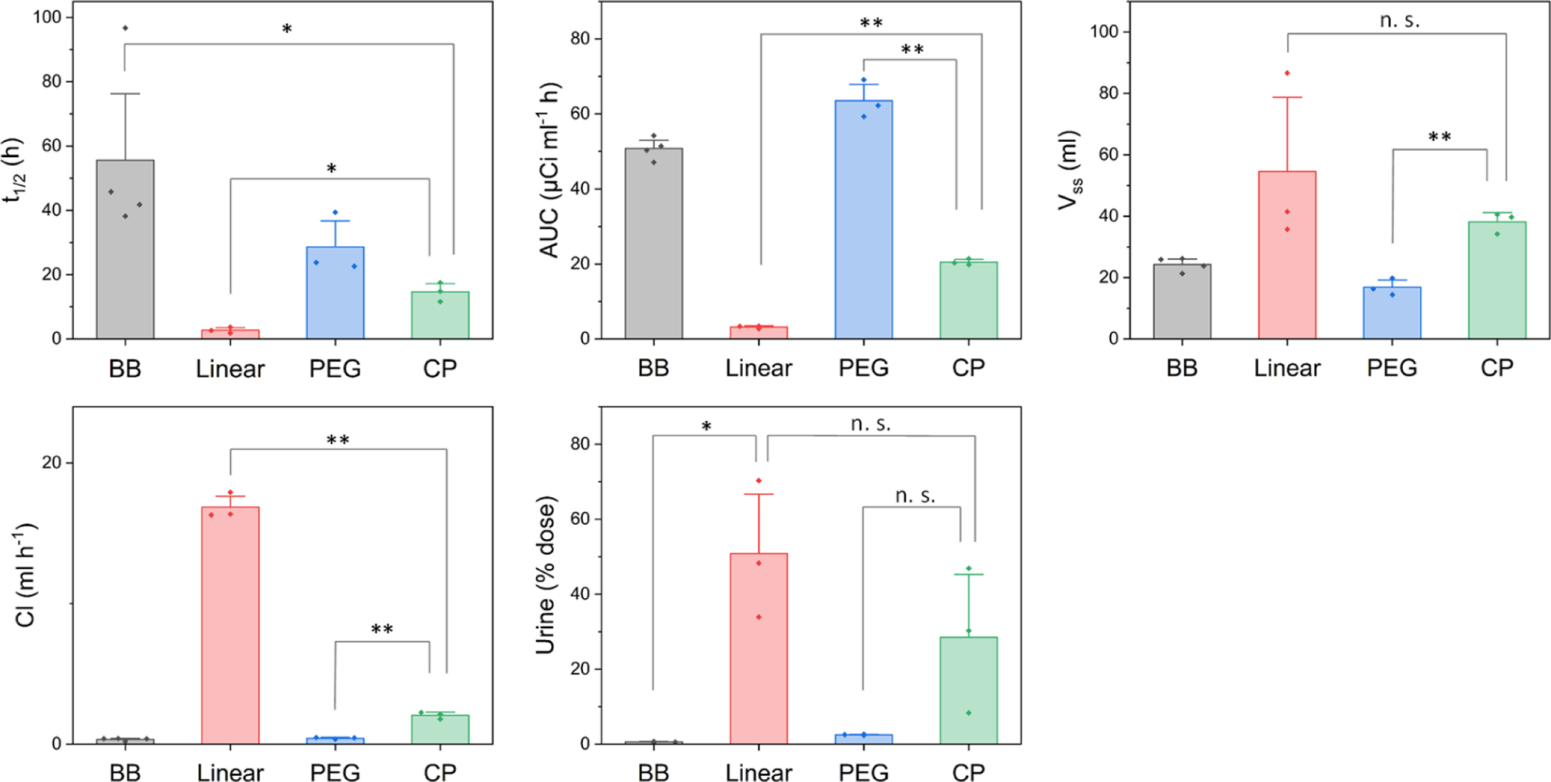
Pharmacokinetic parameters, as listed in Table 2, visualized in the bar chart format. (A) Half-life time, (B) AUC, (C) volume of distribution (steady state), (D) clearance rate, and (E) dose recovered in urine. Error bars indicate standard error. Statistical analysis with the Student *t* test: *p < 0.05 and **p < 0.005.

**Figure 11:**
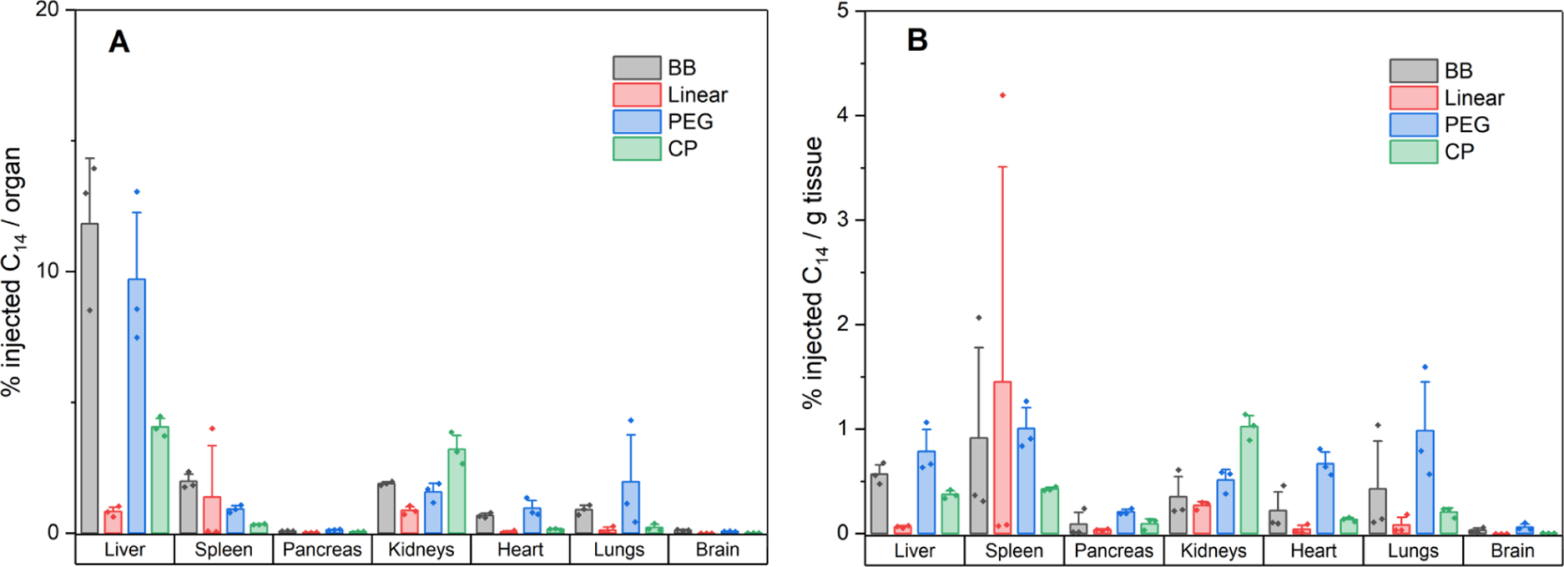
Biodistribution data in major organs of the five compounds determined after 24 h IV dosage. Blank organs were spiked to determine counting efficiencies. Left—% dose found in each organ. Right—% dose per gram tissue of each organ.

The biodistribution of the materials in major organs was determined by harvesting tissues post 24 h dosing and measuring the residual levels of ^14^C radiolabel (Figure 10). The level of accumulation for the linear polymer was very low in all organs (<1.38% dose) (correlating with the high dose recovery in urine) and suggests that rapid renal clearance reduced distribution into organs. Slightly higher values were observed for the **CP**, particularly for the liver and kidneys (4.06 and 3.22% dosage), whereas the BB materials showed the highest organ accumulation, correlating with their longer persistence in the body (i.e., circulation times). The results are also consistent with the suggestion that the self-assembled **CP** nanotube avoids the initial rapid clearance by virtue of its large hydrodynamic volume, but that gradual disintegration into smaller nanotubes/unimers prevents long-term accumulation in organs and ultimately leads to renal excretion. This demonstrates the inherent degradability advantage of the self-assembled CP system, as opposed to the covalently bound BB approach (Figure 11).

The higher accumulation in the liver (and spleen) is indicative of uptake by cells of the mononuclear phagocytic system (MPS), which typically occurs for larger nanoparticles and explains the increased accumulation for **PEG** and **BB** over the other compounds due to their higher MWs.^64^ Similar results for biodistribution of high aspect ratio BBs was reported by Müllner et al.^23^ and suggests that the usage of shorter BBs for drug delivery may be superior than higher aspect ratio materials^65^ as almost identical plasma residence times were obtained without extensive liver accumulation. Comparable biodistribution was observed for the **BB** and **PEG** systems, indicating similar in vivo behavior for both PNAM/PEG materials, although there may be a MW dependence on biodistribution behavior. Figure 10B shows the % dose per gram tissue, which reveals that the BBs have a high affinity for accumulation into both liver and spleen, the latter likely as a result of efficient splenic filtration via MPS clearance. This effect is consistent with a correlation between MW and the spleen uptake, as previously observed for PEGylated nanomaterials.^66^

## Conclusions

Cyclic peptide PNAM conjugates that assemble into 36 nm nanotubes in solution were compared to an equivalent covalently bound BBs of 40 nm in length. Confocal microscopy found evidence of the endosomal uptake, corroborated by a low degree of cell association at 4 °C when compared to the increased values at 37 °C. **CP** possessed a similar uptake to a linear polymer, while higher than the BB materials and so may have advantages in the cellular delivery. Stacking of the **CP** into nanotubes in vivo is the most likely explanation for the increased plasma residence times observed over a comparative MW linear polymer; however, lower half-life times were found for the **CP** than the **BB**, suggesting that the nanotube is either significantly smaller than 40 nm in vivo or that after dynamic self-assembly there is some degree of degradation. This is reflected in the biodistribution results where the **CP** shows reduced organ accumulation, likely due to the ability to disassociate into low MW unimers. The ability of **CP** delivery systems not only to circulate longer than their linear comparators but also to clear readily via disassembly may provide advantages for drug delivery to tumors where initial accumulation is desirable but often smaller polymer/nanoparticles are found to penetrate tissues more effectively, or for imaging of tumors where a “fast in-fast out” profile is required to reduce the background. Additionally, a PEG BB was studied to explore the potential use of PNAM as a biocompatible drug delivery material. The PNAM brush was found to perform comparably with plasma half-lives exceeding 35 h. The ease and flexibility of synthesis of NAM polymers by RAFT make these appealing candidates for further research. Future investigations will study the behavior of the CP and PNAM brushes in tumor models to further assess their potential in anti-cancer treatments.

## Supporting Information Available

Polymer brushes and control polymer syntheses; kinetics of polymerizations and material analyses and characterization (matrix-assisted laser desorption ionization, NMR, gel permeation chromatography, AFM, fluorescence, and HPLC); confocal microscopy of MDA cells; and uncorrected pharmacokinetic plasma concentrations (PDF)
