# Supplementary material for: Polymeric Nanotubes as Drug Delivery Vectors—Comparison of Covalently and Supramolecularly Assembled Constructs
Source: Biomacromolecules. 2022 May 18;23(6):2315–28. doi: 10.1021/acs.biomac.2c00063 (PMC9198979; doi:10.1021/acs.biomac.2c00063)
Supplement: Supplementary file 1 — bm2c00063_si_001.pdf [file bm2c00063_si_001.pdf]

Supporting Information

Polymeric nanotubes as drug delivery vectors – comparison of a covalently and supramolecularly assembled construct

*Andrew Kerr,[a] Erny Sagita,[b] Edward D. H. Mansfield,[a] Tri-Hung Nguyen,[b] Orlagh Feeney,[b] Colin W. Pouton,[b] Christopher J.H. Porter,[b] Joaquin Sanchis, *[b] and Sébastien Perrier.*[a,b,c]*

a Department of Chemistry, The University of Warwick, Coventry CV4 7AL, UK

b Drug Delivery Disposition and Dynamics, Monash Institute of Pharmaceutical Sciences, Monash University, 381 Royal Parade, Parkville, VIC 3052, Australia

c Warwick Medical School, The University of Warwick, Coventry CV4 7AL, UK

Materials

4-acryloylmorpholine (NAM, 97%) was obtained from Sigma-Aldrich and passed through a basic alumina column before use. N-Acrylic acid hydroxysuccinimide ester (NAS, >90%), 4,4′-azobis(4-cyanovaleric acid) (ACVA, >98%), acryloyl chloride (>97%), acetonitrile, trimethylamine, diisopropylethylamine (DIPEA), triisopropylsilane, dimethyl sulfoxide-d_6 (99.9% D atom) and chloroform-*d* (99.8% D atom) were obtained from Sigma Aldrich and used as received. CH_3O-PEG-NH_2 (MW 2000 Da, Rapp Polymere), Alexa-488 Cadaverine (Fisher), ^{14}C-Ethanolamine (50-60 μCi/mmol, American Radiolabeled Chemicals), 1,4-dioxane (anhydrous, Acros Organics), N,N-dimethylformamide (anhydrous, Acros Organics), N-methylmorpholine (NMM, Alfa Aesar) and piperidine (Alfa Aesar) were used as received. 4-(4,6-Dimethoxy-1,3,5-triazin-2-yl)-4-methylmorpholinium tetrafluoroborate ($DMTMM{\cdot}BF_4$), O-(1H-6-Chlorobenzotriazole-1-yl)-1,1,3,3-tetramethyluronium hexafluorophosphate (HCTU), 2-chlorotrityl resin, Fmoc-D-Leu-OH, Fmoc-L-Lys(Boc)-OH, Fmoc-L-Trp(Boc)-OH were purchased from Iris Biotech and used as received.

Small angle neutron scattering

Prior to measuring, all samples were dissolved in D_2O to a concentration of 5 mg/mL, apart from CP-POX which was dissolved at 2.5 mg/mL due to poor solubility at higher concentrations, before being placed in 2 mm quartz cuvettes and placed in the sample holder. Each raw scattering data set was corrected for the detector efficiencies, sample transmission and background scattering and converted to scattering cross-section data ($\partial\Sigma/\partial\Omega$ vs. Q) using

the instrument-specific software.[1] These data were placed on an absolute scale (cm^{-1}) using the scattering from a standard sample (a solid blend of hydrogenous and perdeuterated polystyrene) in accordance with established procedures.[2]

To model the data, the SASfit software package was used, with the (CYL+Chains_RW) model which uses the following form factor:

$$P(q) = N^2\beta_s^2 F_{s(q)} + N\beta_c^2 F_{c(q)} + 2N^2\beta_s\beta_c S_{sc(q)} + N(N-1)\beta_c^2 S_{cc(q)}$$

where N is the aggregation number, $\beta_s = V_s\,(\rho_s - \rho_{solv})$ and $\beta_c = V_c\,(\rho_c - \rho_{solv})$ are the total excess scattering lengths of a block in the cylindrical core and in the corona, respectively. V_s and V_c are the volumes of a block in the core and in the corona, respectively. ρ_s and ρ_c are the corresponding scattering length densities and ρ_{solv} is the scattering length density of the surrounding solvent.

$F_{s(q,R,L)} = F_{cs(q,R)} F_{L(q,L)}$ where $F_{cs(q,R)} = \left[\frac{2B_1(qR)}{qR}\right]^2$, $F_{L(q,L)} = 2\frac{Si(qL)}{qL} - \frac{4sin^2\left(\frac{qL}{2}\right)}{q^2L^2}$

B_1 is the first order Bessel function and $Si(x) = \int_0^x \frac{\sin t}{t} dt$

$$F_{c(q)} = \frac{2\left[\exp(-q^2R_g^2) - 1 + q^2R_g^2\right]}{q^4R_g^4}$$

$S_{sc(q)} = \psi(qR_g)\frac{2B_1(qR)}{qR} B_0\left[q(R + dR_g)\right] F_{L(q,L)}$ where $\psi(qR_g) = \frac{1-exp(-qR_g)}{qR_g}$, R_g is the gyration radius of the block of the corona, and B_0 is the zero[th] order Bessel function.

$$S_{cc(q)} = \psi(qR_g)^2 B_0\left[q(R + dR_g)\right]^2 F_{L(q,L)}.$$

For the cylindrical micelle model, SLD values for the solvent, peptide core, and polymer corona were calculated using based on the molecular structure. Vbrush (the molecular volume of the polymer arms) was calculated by dividing the Mw of the polymer by Avogadro's number multiplied by the density. In all cases the R_core value was fixed at 5 Å (representing the radius of the cyclic peptide itself), d was fixed at 1 (to mimic the non-penetration of chains into the cylindrical core of the peptide), and xsolv was set to 0. The remaining parameters (N, n_agg, Rg and H) were left as floating variables during the fitting process.

Synthesis of linear peptide

A previously described literature procedure was followed.[3] Synthesis of NH_2-L-Lys(Boc)-D-Leu-L-Trp(Boc)-D-Leu-L-Lys(Boc)-D-Leu-L-Trp(Boc)-D-Leu-COOH was performed using

a Prelude (Protein Technologies inc.) automated solid phase peptide synthesiser, using a previously described procedure. 2-chlorotrityl resin (0.36 g) was allowed to swell with DCM prior to loading by addition of a solution of Fmoc-D-leu-OH (1.01 g, 2.86 mmol) and DIPEA (0.4 M) in DMF (16 ml) and reacted for 2 h, drained and then treated with DCM / MeOH / DIPEA (17:2:1, 10 ml) to ensure capping of unreacted resin sites. The drained resin was washed with DMF, and 20% Pipierdine solution in DMF (15 ml) was added to deprotect the Fmoc groups, followed by further washing with DMF. Subsequent coupling steps were performed by addition of Fmoc-amino acid (2.86 mmol) with an HCTU (0.83 g, 0.20 mmol) and NMM (0.44 ml, 0.4 mmol) solution in DMF (10 ml), left to react for 2 h and then washed with DMF. Further deprotection and addition steps were repeated until the targeted octapeptide was synthesised. After the final Fmoc deprotection step the peptide was cleaved from the resin by addition of HFIP (20 %) in DCM (3 x 10 ml) and washed with DCM. The solution was concentrated under vacuum to yield an off-white solid. ESI MS +ve: Calcd for $[M+Na]^+$ 1503.89. m/z 1503.8 found.

Synthesis of PNAM-co-PNAS side chain bottle-brushes

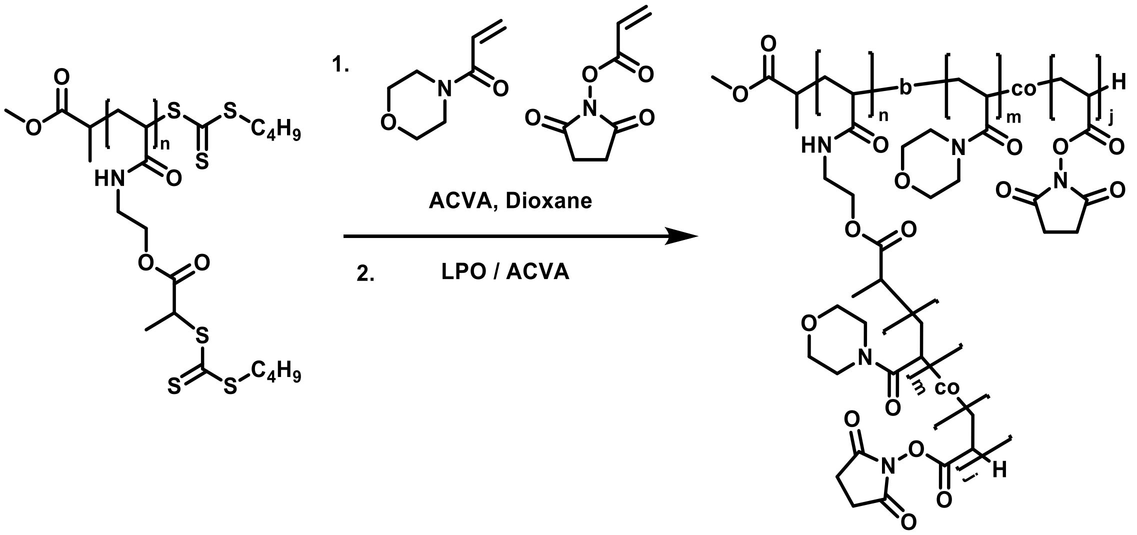

PolyCTA (11.9 mg, $3.45*10^{-2}$ mmol, 1 eq.), NAM (1g, 7.08 mmol, 200 eq.), NAS (60 mg, $3.45*10^{-1}$ mmol, 10 eq.), ACVA (0.66 mg, $2.36*10^{-3}$ mmol) and anhydrous dioxane (2.8 ml) were added to a vial fitted with a stirrer bar and rubber septum. The reaction mixture was degassed with nitrogen for 10 minutes and placed in an oil bath heated to 60°C for 2h. The monomer conversion was determined by ^1H NMR, the polymerisation was stopped at approximately 25 % conversion to target a DP of 50 for the side chains. The reaction mixture was precipitated 3 times into ice cold diethyl ether and dried under vacuum to yield a pale yellow powder.

The (PNAM$_{50}$-co-PNAS$_{2.5}$) bottle brush (300 mg, 4.0*10^{-2} mmol CTA), ACVA (336 mg, 1.2 mmol, 30 eq.), Lauroyl peroxide (47.8 mg, 0.12 mmol, 3 eq.) were dissolved in anhydrous dioxane (15 ml), degassed with nitrogen for 10 minutes and heated in an oil bath at 80°C for 6 h. The reaction mixture was precipitated 3 times into ice cold diethyl ether, then redissolved in water and dialysed for 3 days against a 10k MWCO membrane. The aqueous solution was freeze dried to yield a colourless powder. SEC analysis confirmed removal of the characteristic UV absorption at 309 nm of the trithiocarbonate groups. ^{1}H NMR (300 MHz, $CDCl_3$) δ 4.0 – 3.0 (8H, (OC<u>H</u>$_2$C<u>H</u>$_2$N)$_2$), 2.81 (4H, C(O)C<u>H</u>$_2$C<u>H</u>$_2$C(O)), 2.6 -2.2 (1H, NC(O)C<u>H</u> (NAM)), 2.0 – 1.0 (3H, C<u>H</u>$_2$C<u>H</u> backbone).

Synthesis of linear PNAM-co-pNAS

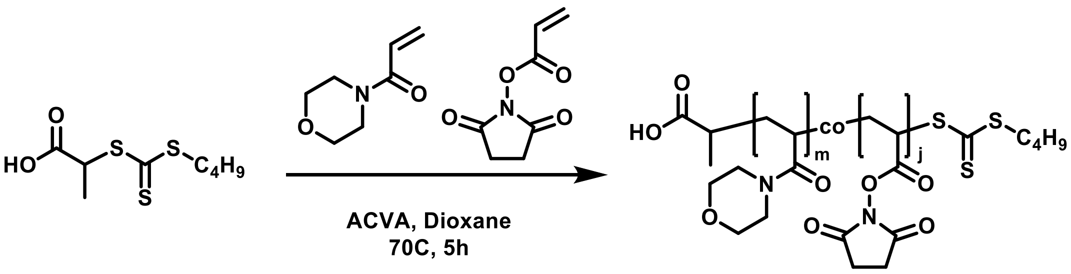

NAM (200 mg, 1.42 mmol, 100 eq.), Acrylic acid NHS (12 mg, 7.08*10^{-2} mmol, 5 eq.), PABTC (3.4 mg, 1.42*10^{-2} mmol. 1eq.) and ACVA (0.79 mg, 2.83*10^{-3} mmol) were dissolved in 0.53 ml anhydrous dioxane in a 3 ml vial fitted with a stirrer bar and rubber septum. The reaction mixture was degassed with nitrogen for 10 minutes, placed in oil bath set to 70°C for 5h and then precipitated twice into diethyl ether, dried under vacuum to yield a pale yellow powder.

Synthesis of PEG acrylamide macromonomer

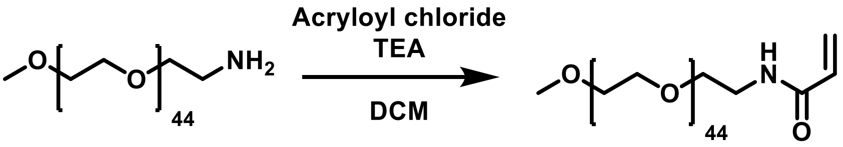

Amino-PEG (4.5 g, 2.25 mmol, M_n = 2,000 g mol^{-1}) was dissolved in 60 ml anhydrous DCM in a 250 ml round bottom flask under nitrogen, triethylamine (364 mg, 3.60 mmol) was added and then the reaction mixture was cooled with an ice bath. Acryloyl chloride (306 mg, 3.375 mmol) was added dropwise to the reaction mixture, after 2 h the ice bath was removed and left to stir overnight at room temperature. The DCM was concentrated under vacuum, the residue redissolved in 0.1 M $NaHCO_3$ solution (30 ml) and extracted twice with DCM. The organic layer was collected, dried, over $MgSO_4$, filtered and concentrated. The crude mixture was then

purified by passing through a silica plug using DCM / MeOH 9:1 as the eluent, the collected fractions were concentrated to yield a white powder (3.5 g, 78 % yield). . ^{1}H NMR (300 MHz, $CDCl_3$) δ 6.21 (dd, J = 9.6 Hz, 17.1 Hz, 1H), 6.12 (dd, J = 1.8 Hz, 17.1 Hz, 1H), 5.69 (dd, J = 1.8 Hz, 9.3 Hz, 1H), 3.63 (s, 1H), 3.40 (m, 2H), 3.30 (s, 3H). MALDI analysis confirmed addition of vinyl end group.

Synthesis of PEG bottle-brushes

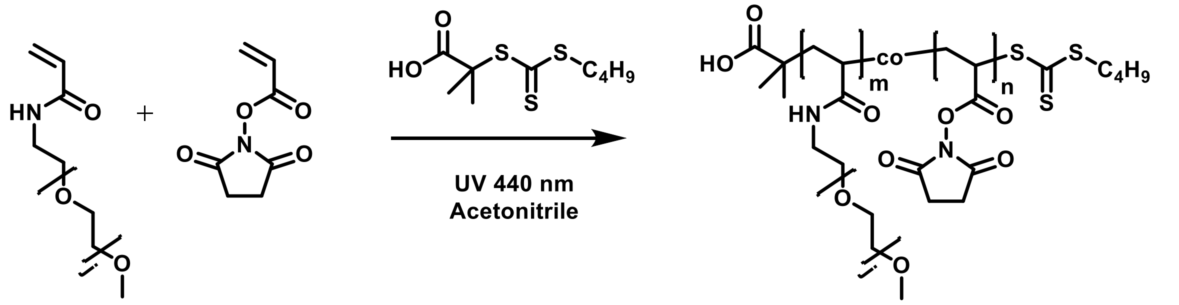

PEG acrylamide (100 mg, $5*10^{-2}$ mmol), BDMATC (0.63 – 0.08 mg, depending on target DP) and NAS (0.8 mg, $5*10^{-3}$ mmol) were dissolved in 300 µl acetonitrile, degassed with nitrogen and placed in a UV curing box (λ = 440 nm) for 48 h. The macromonomer conversion was monitored by size exclusion chromatography reaching >90 %. The reaction mixture was then precipitated three times into ice cold diethyl ether to remove residual macromonomer and dried under vacuum to yield a colourless powder.

Fluorescence labelling by conjugation of Alexa-488 dye

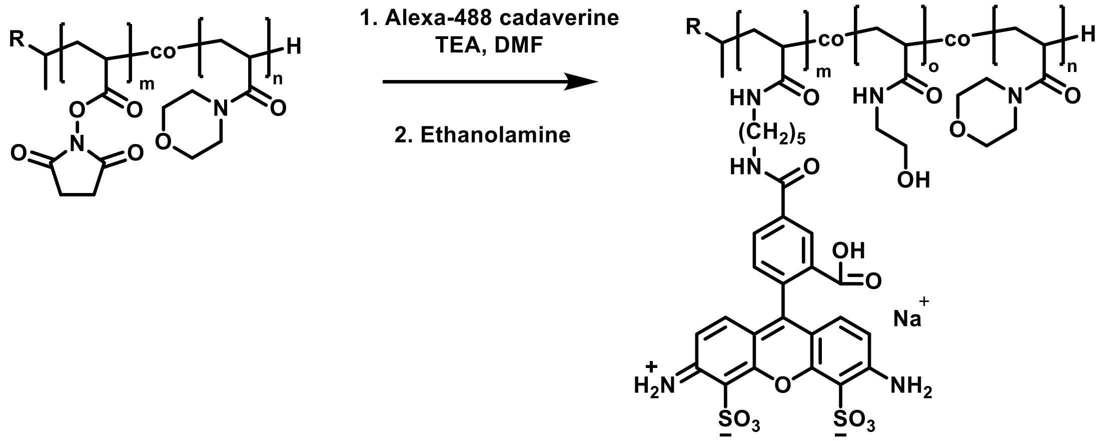

The PNAM-co-PNAS short brush (10 mg, $3.33*10^{-3}$ mmol NAS units), TEA (1 mg, 9.88*10-3 mmol) and Alexa-488 cadaverine (0.03 mg, $4.68*10^{-5}$ mmol) were dissolved in 250 µl anhydrous DMF in a 1.5 ml screw cap vial and stirred for 2 days at room temperature in the dark. After which ethanolamine (2 µl, $3.27*10^{-2}$ mmol) was added and stirred for a further 4 h

at room temperature, after which the DMF was removed under a stream of nitrogen, the residue redissolved in water and immediately passed through a Sephadex PD10 column. The fractions were analysed by fluorescence spectrometer to reveal full conjugation of the dye onto polymer. The polymeric fractions were combined, washed three times through a centrifuge dialysis tube (100k MWCO) and freeze dried to yield the compound as a colourless powder.

The same procedure was used for the other polymers, except for the linear in which a lower 10k MWCO of the dialysis centrifuge tube was used. For the cyclic peptide conjugate a different ratio of reagents was used: CP-NAS extended (3 mg, $2.74*10^{-3}$ mmol NAS units) and Alexa-488 cadaverine (0.09 mg, $1.41*10^{-4}$ mmol), otherwise the same reaction procedure was carried out.

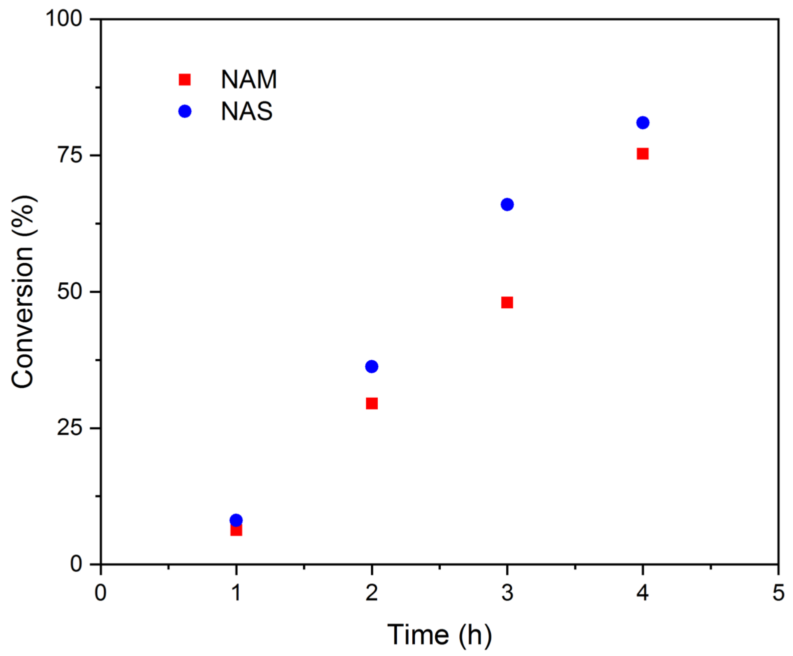

Figure S1: Kinetics of a NAM / NAS copolymerisation showing similar rate of consumption of both types of monomer, consistent with literature desribing their similar reactivity ratios.

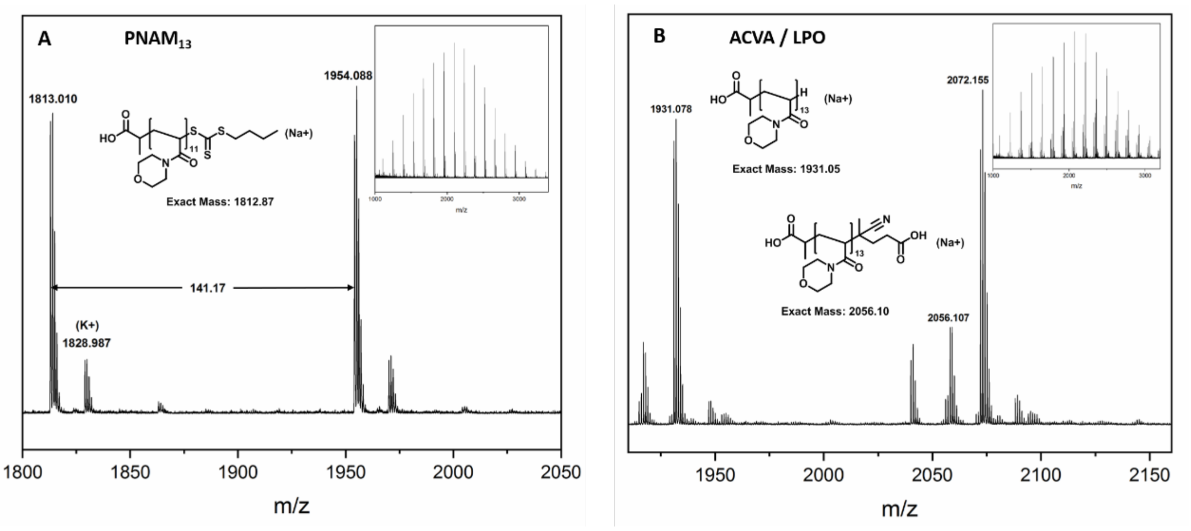

Figure S2: MALDI mass spectra of: **A** - PNAM$_{13}$ polymer synthesised with a RAFT agent end group, **B** – PNAM$_{13}$ polymer post reaction with ACVA / LPO revealing full removal of the RAFT end group. This MALDI analysis of a test linear PNAM treated under the same revealed full removal of the trithiocarbonate end group and formation of predominately 'H' terminated chains, with a small number of possible ACVA end groups, although MALDI is not a quantitative technique for the determination of the relative composition of different species. The end groups of the bottle-brush side chains will be altered in the same manner.

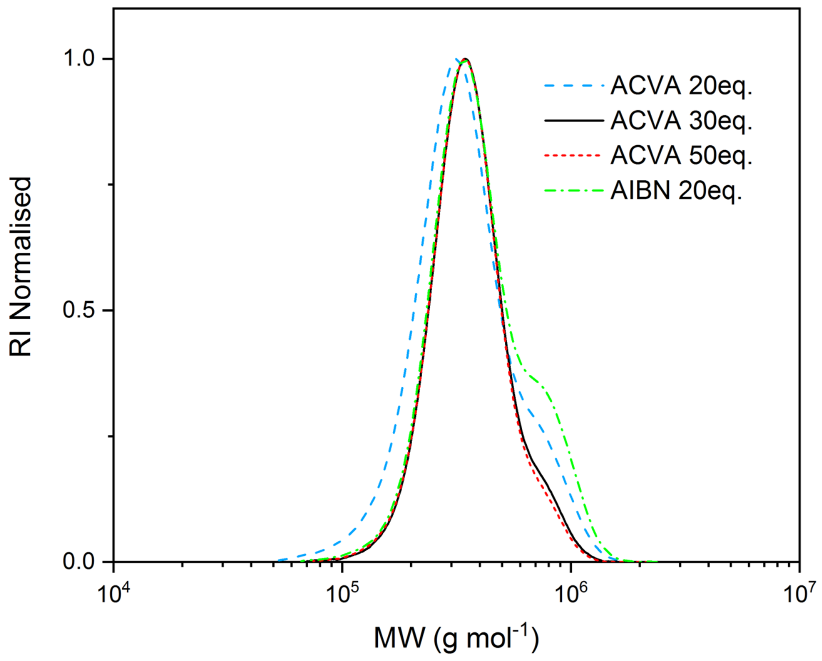

Figure S3: SEC molecular weight distributions of the CTA removal step performed on the PNAM bottle-brush compound with various reaction conditions. Formations of a high molecular weight shoulder is reduced by using a larger excess of ACVA azoinitiator. AIBN was also tested to conifrm the trend was not limited to the use of ACVA in particular.

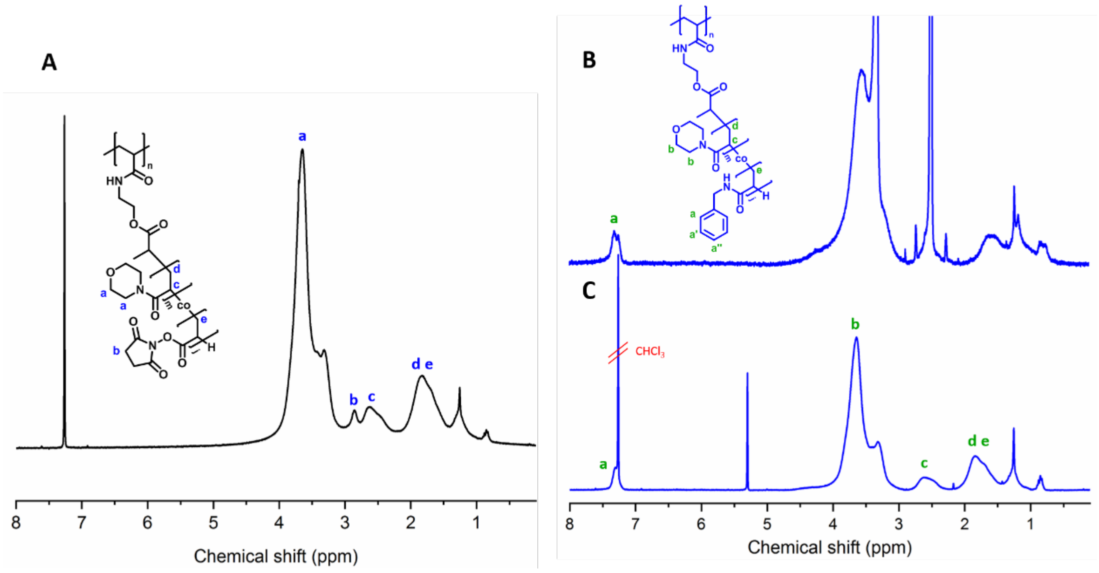

Figure S4 A – ^{1}H NMR spectrum in $CDCl_3$ of the purified PNAM-co-PNAS bottle-brush. **B** – ^{1}H NMR spectrum in $CDCl_3$ after reaction with benzylamine displaying full consumption of the NHS ester environments. **C** – ^{1}H NMR spectrum in DMSO-d_6 of the benzylamine conjugate showing presence of aromatic signals. To probe efficacy of the amide coupling reaction, firstly the **BB** was treated with a 2 equivalents excess of benzylamine in DMF with TEA, whereby

after 24 h reaction time ^{1}H NMR revealed full consumption of the NHS ester peak at 2.85 ppm and in DMSO-d_6 the introduction of the benzyl group onto the polymer with peaks at ~7.2 ppm is clearly observed (B, C). Thus the coupling readily occurs to high yield at room temperature and is a suitable approach for introduction of labelling moieties.

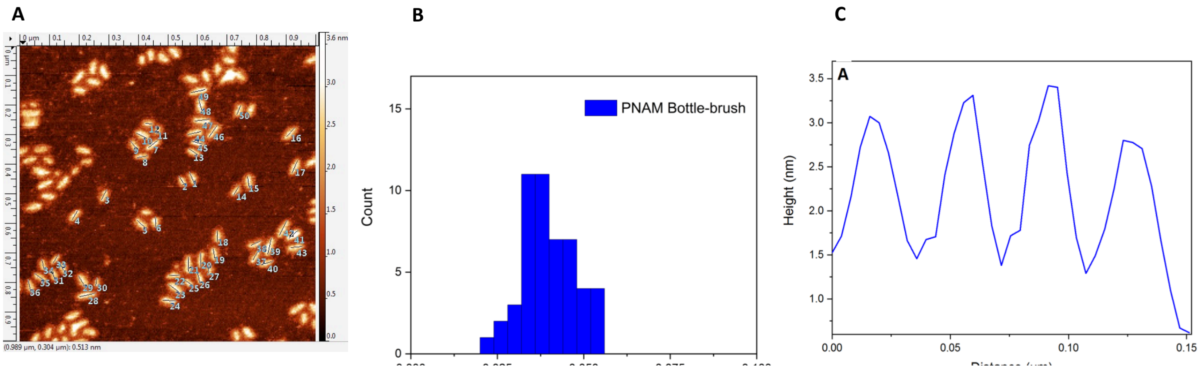

Figure S5: The PNAM bottle-brush molecular size was determined by measuring the length of 50 molecules by AFM (A, end-to-end distance), the data shown as a histogram in B to give an average of 40 nm. C – Height profile of four bottle-brush moelcules extracted form figure S5-A.

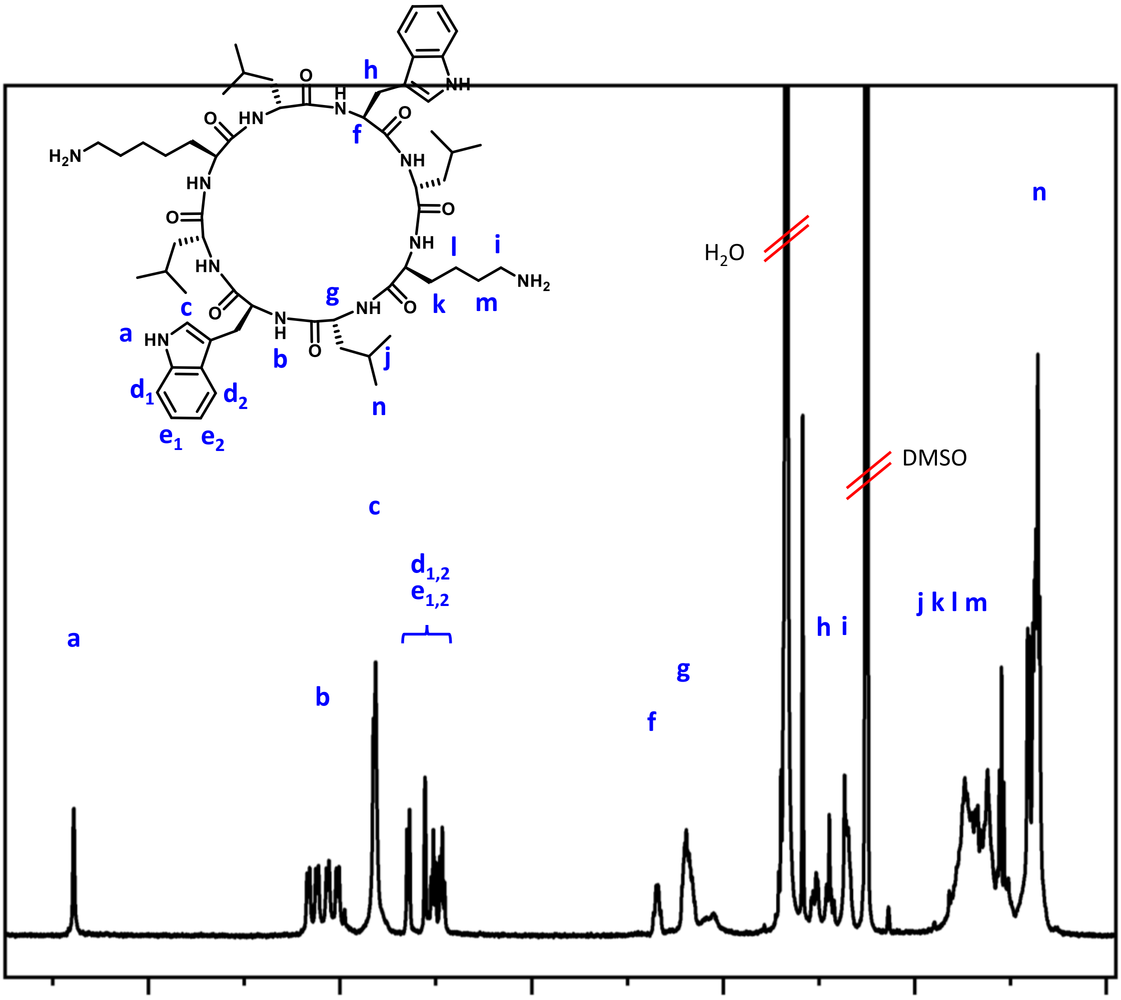

Figure S6: ^{1}H NMR spectrum (400 MHz) of the deprotected cyclic peptide performed in DMSO-d_6.

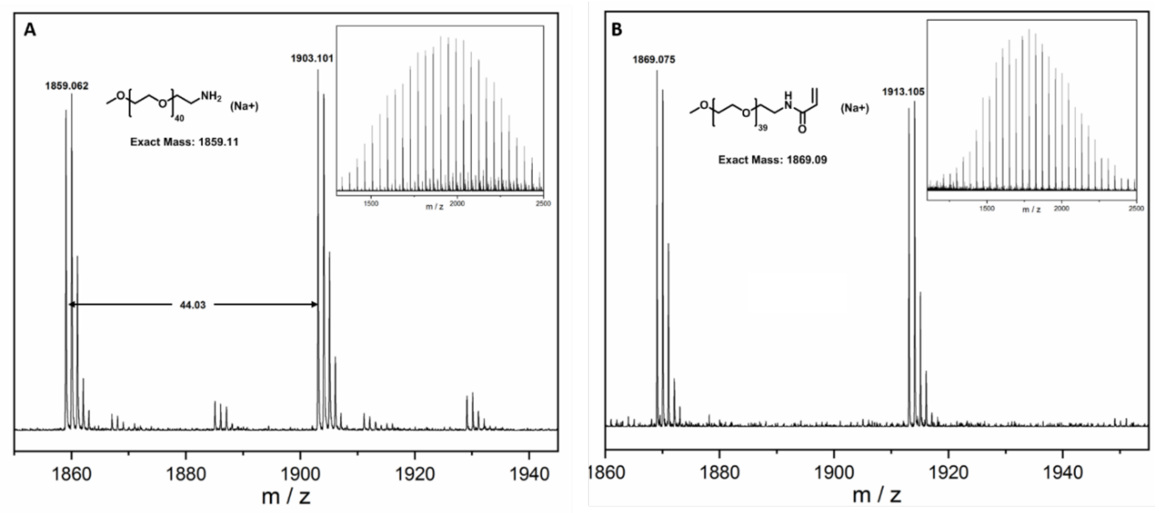

Figure S7: MALDI-ToF spectra of A - the commerical starting material amine terminated PEG (2,000 $gmol^{-1}$) and B – the synthesised PEG macromonomer.

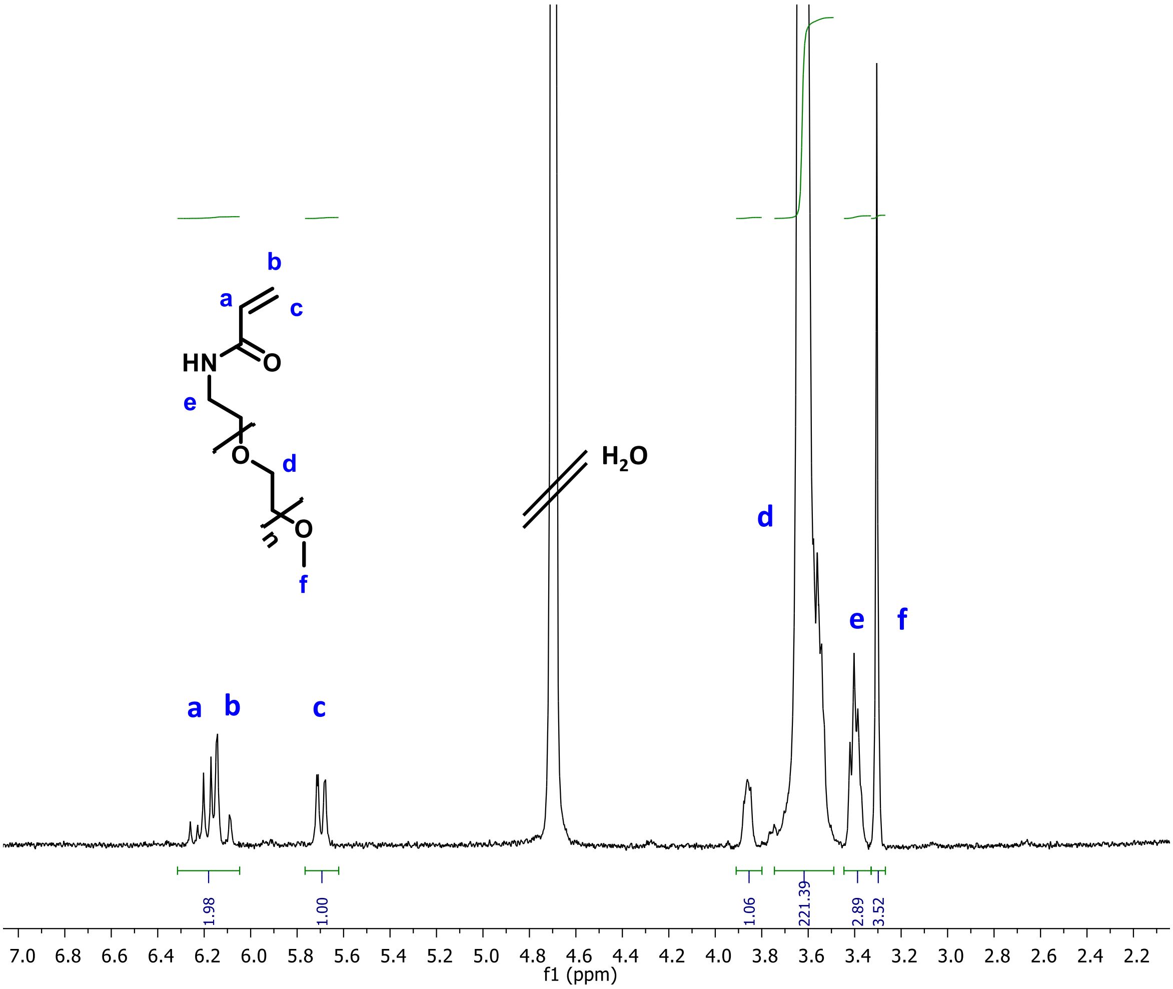

Figure S8: ^{1}H NMR spectrum (300 MHz) of the synthesised PEG macromonomer in $CDCl_3$.

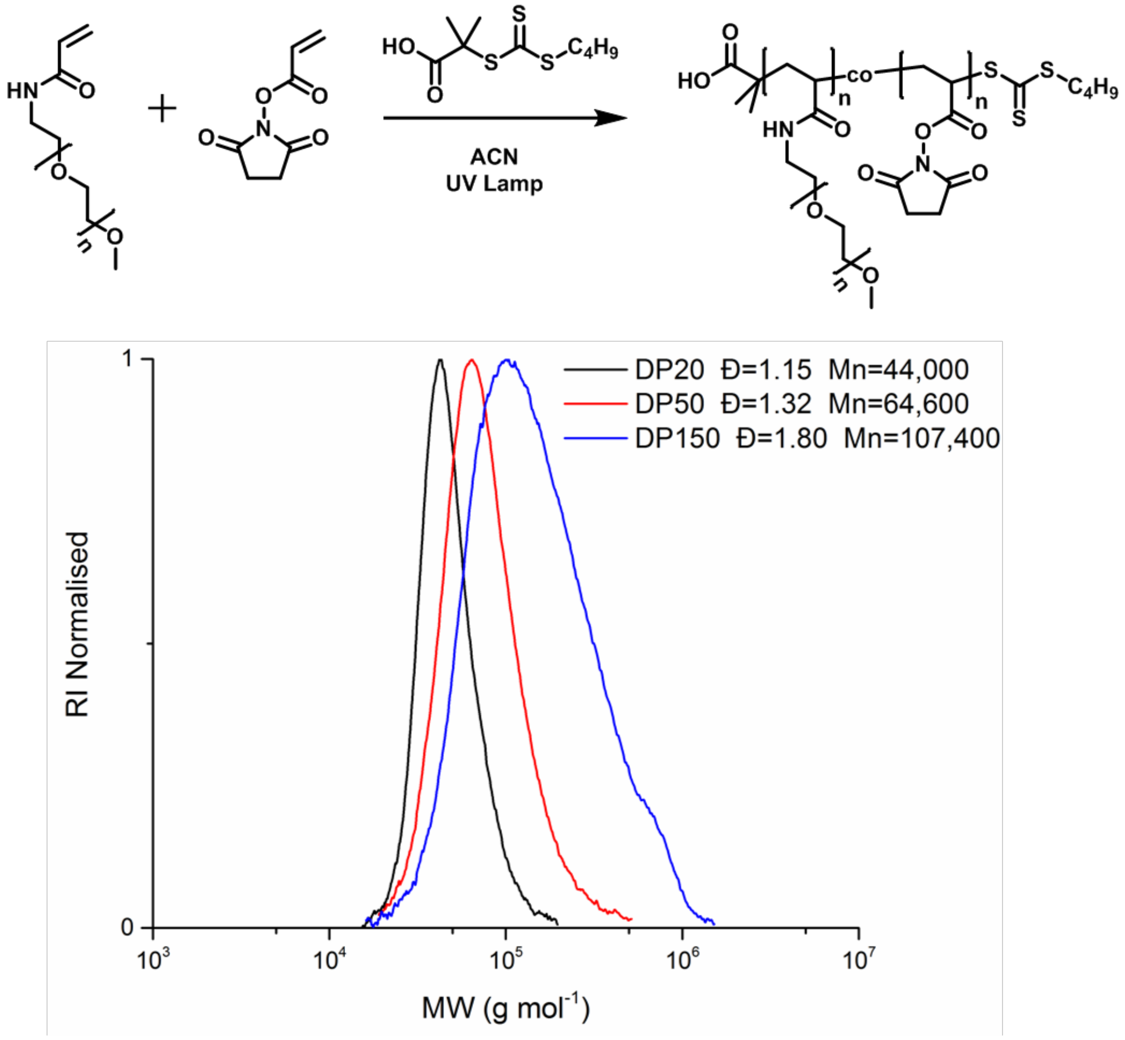

Figure S9: Top – synthetic scheme of the PEG macromonomer polymerisation to from a bottle-brush. Bottom- SEC molecular weight distributions of the PEG brushes targeting 3 different degrees of polymerisation. Above DP50 a significant increase in dispersity was observed.

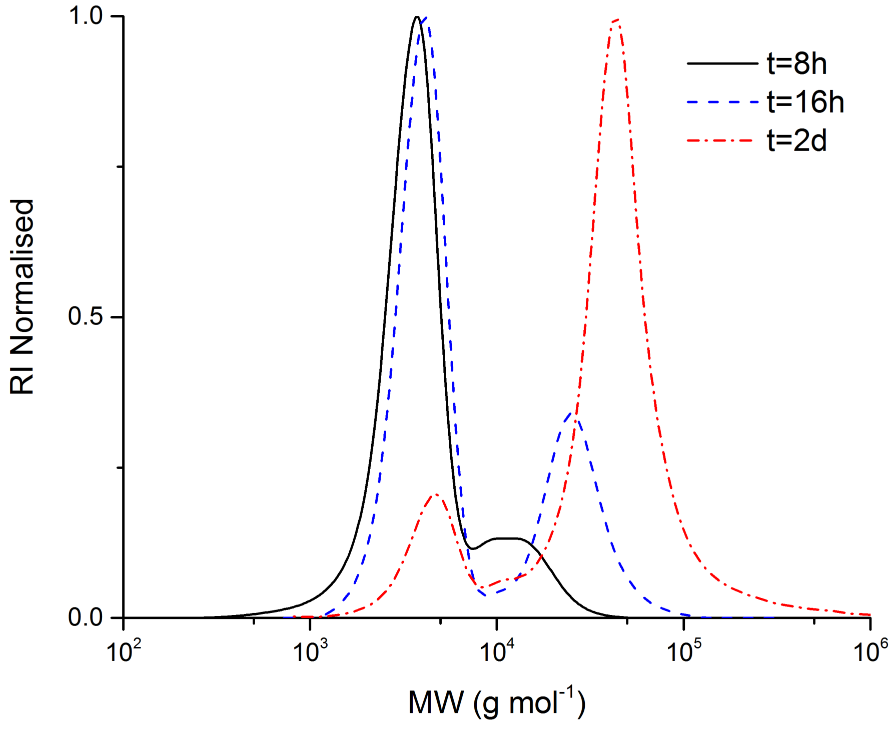

Figure S10: Kinetic SEC analysis of the PEG macromoner polymerisation, used to determine macromonomer consumption by integration of each peak

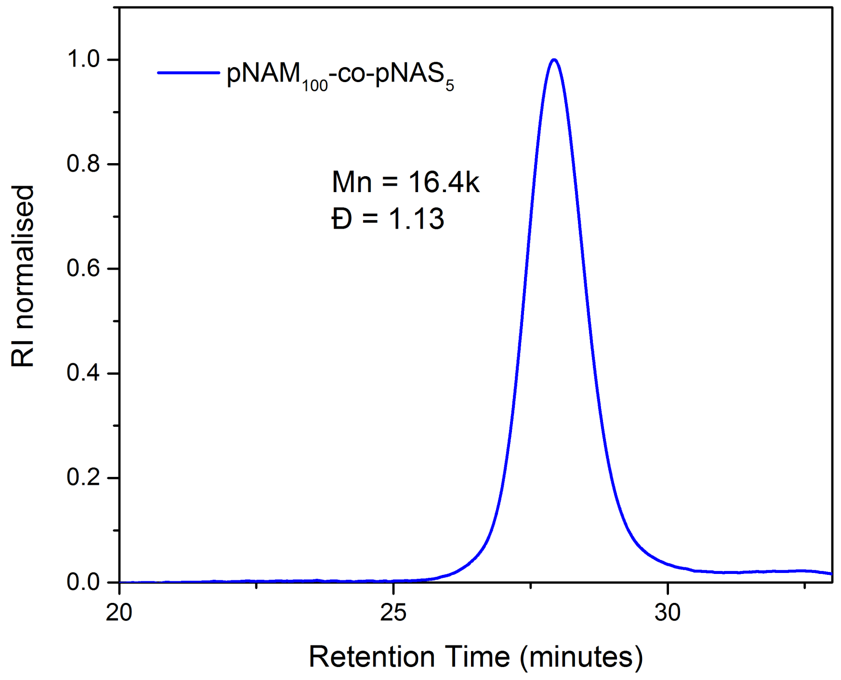

Figure S11: SEC molecular weight distribution of the linear PNAM-co-PNAS polymer, performed on the DMAc eluent SEC system.

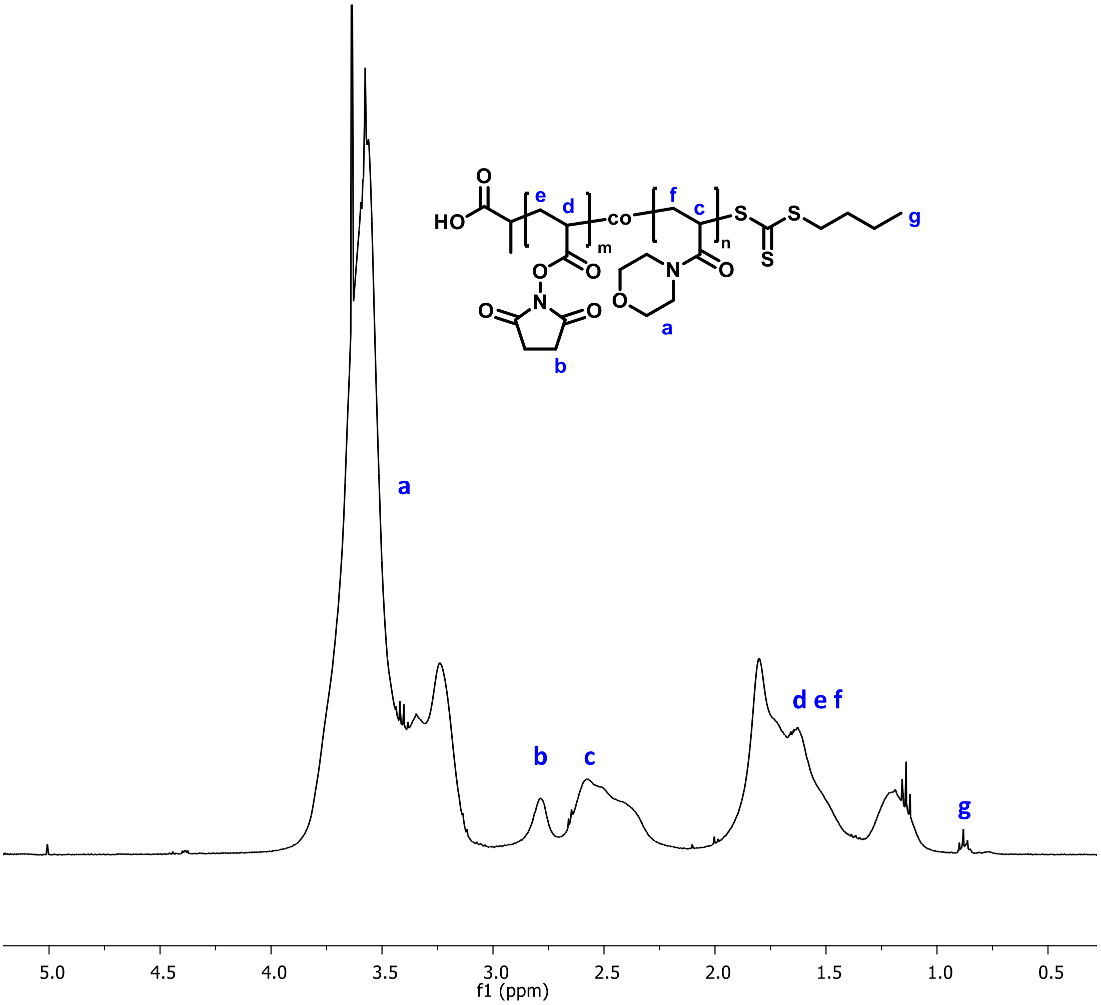

Figure S12: ^{1}H NMR spectrum (300 Mhz) of PNAM-co-PNAS linear polymer compound.

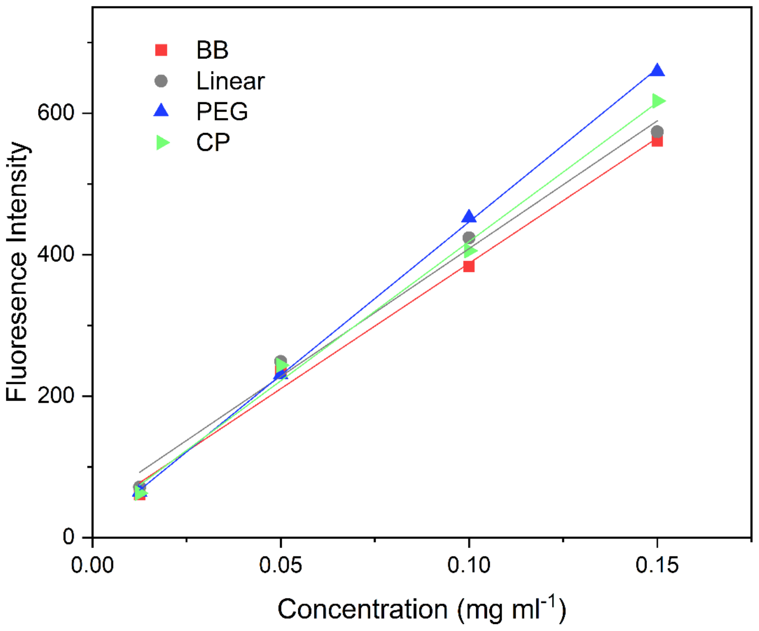

Figure S13: Fluorescence calibration curves for the Alexa-488 labelled materials.

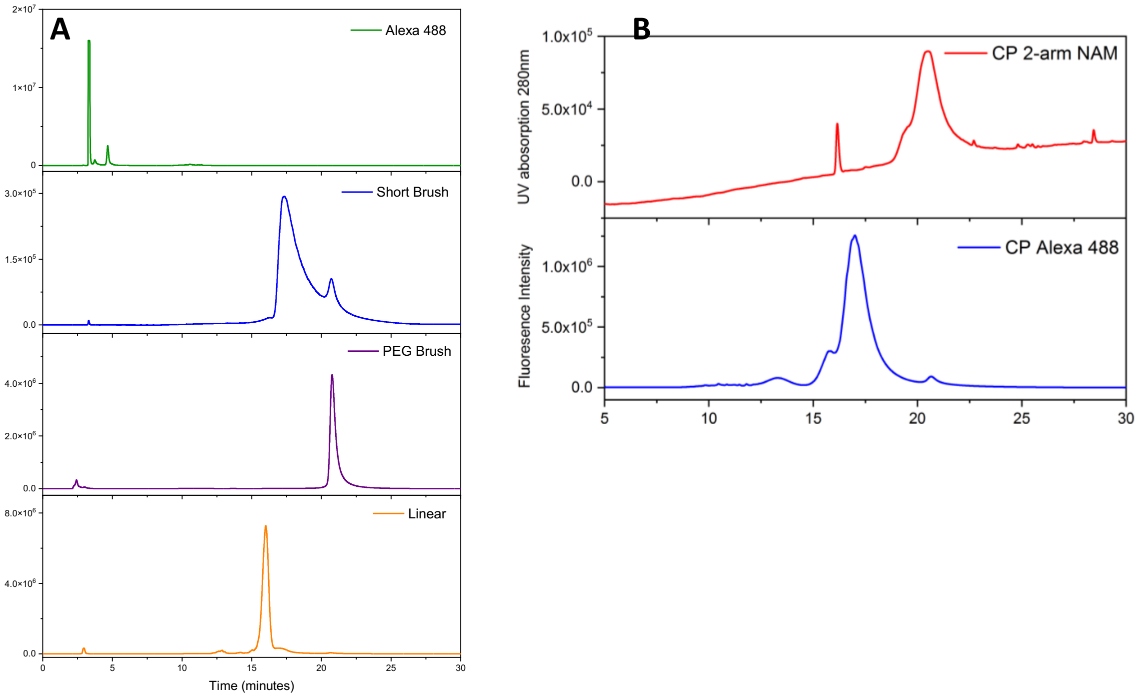

Figure S14: **A** – HPLC traces of polymers after modification with Alexa-488 monitored by fluorescent intensity at the 490 nm / 525 nm excitation/emission wavelengths. **B** – HPLC traces of the CP before and after chain extension and functionalisation with dye.

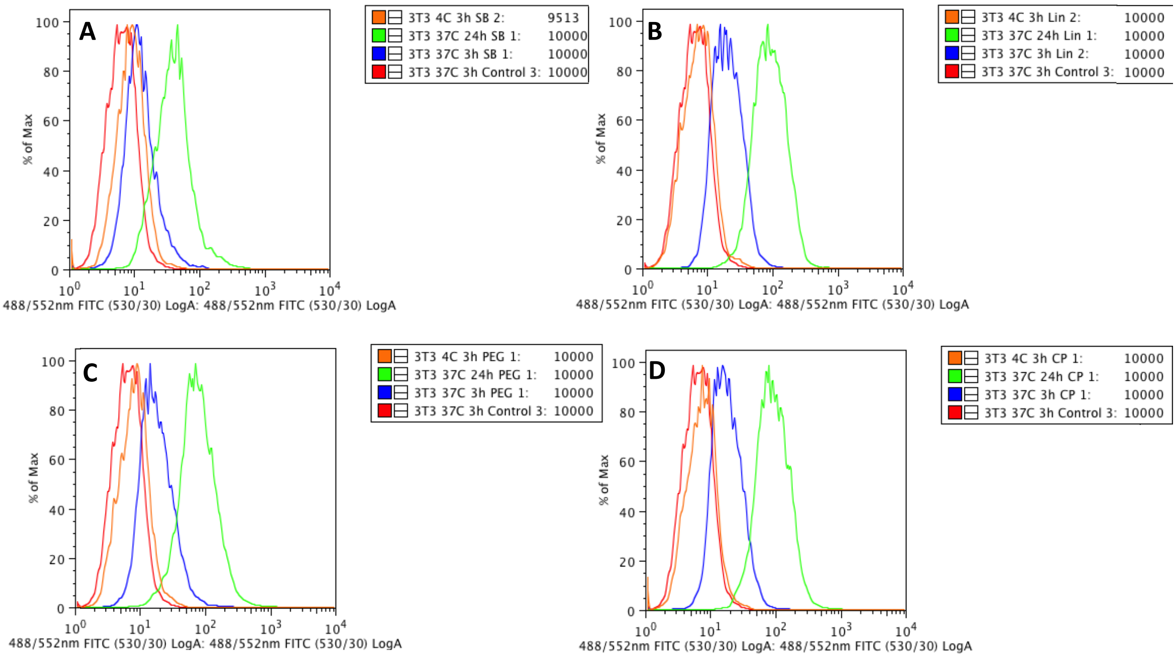

Figure S15: Flow cytometry histograms of the 3T3 cell lines under 4 experimental conditions for the various compounds: **A** – BB, **B** – Linear, **C** – PEG, and **D** – CP.

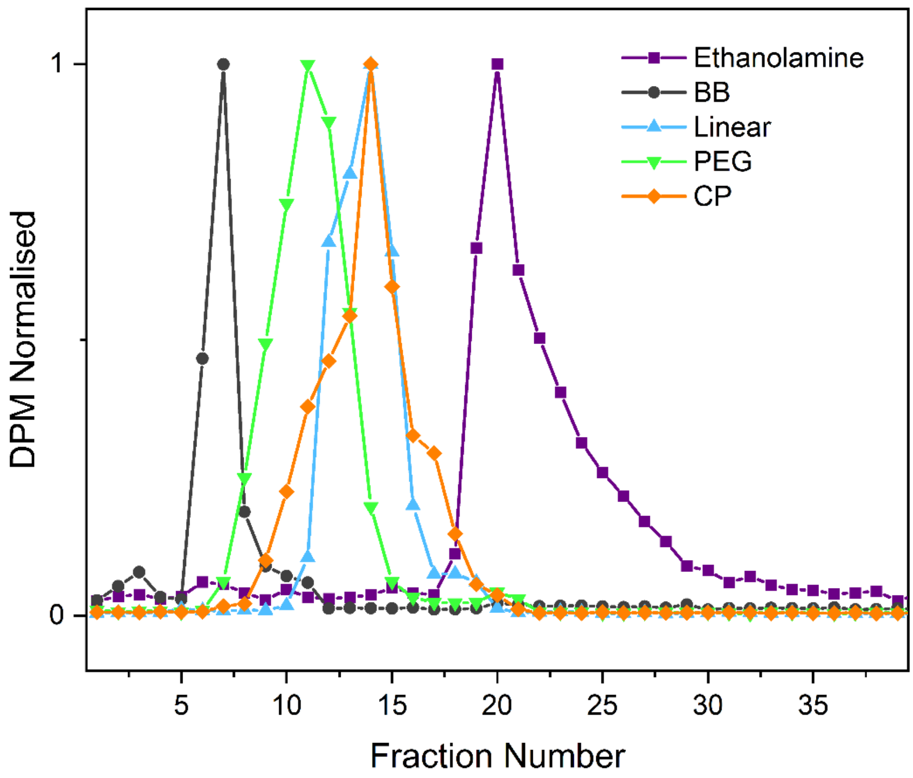

Figure S16: SEC analysis of the purified radiolabelled compounds, showing the radioactivity (DPM) of each fraction measured by scintillation counting. All polymeric formulations do not contain residual free ethanolamine, therefore pharmacokinetic measurements should reliably monitor the polymeric constructs.

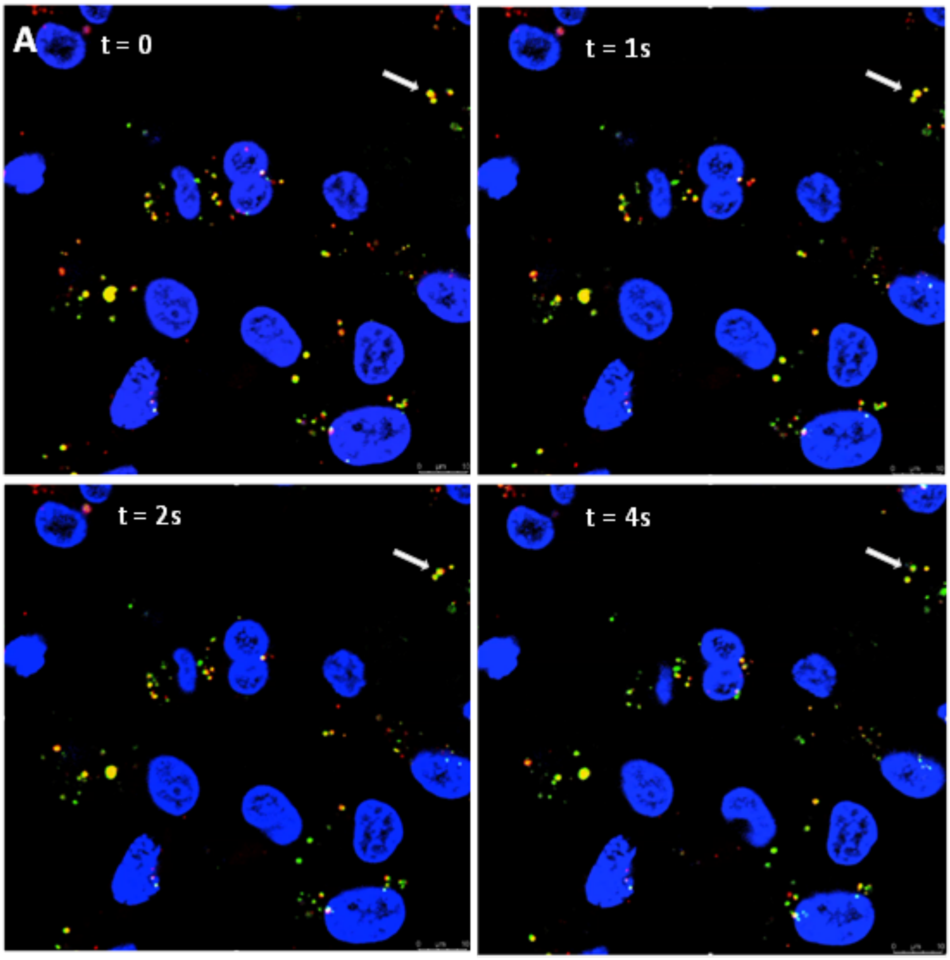

Figure S17: Confocal microscopy showing still images of a time lapse experiment at various time points of MDA cells treated with the dye labelled CP conjugate. At the recorded settings, the resolution of the confocal microscope was approximately 200 nm and, therefore, it is possible the samples are not truly colocalised but rather happen to be within 200 nm distance. To elucidate this, additionally a time-lapse confocal imaging experiment was carried out on the **CP** and **BB** compounds in live cells to clarify coincidence with the lysosome. As demonstrated by still images at various time points, the yellow regions, resulting from stacked compound and lystoracker, remain colocalisied over the course of the time lapse despite motion between frames. This therefore provides further evidence of strong lysosomal compartmentalisation rather than incidental overlay by random fluctuations. It is worth noting after 4 seconds of recording quenching of the red lysotracker dye is observed, causing the red/yellow colour to fade, and therefore longer measurements times could not be performed.

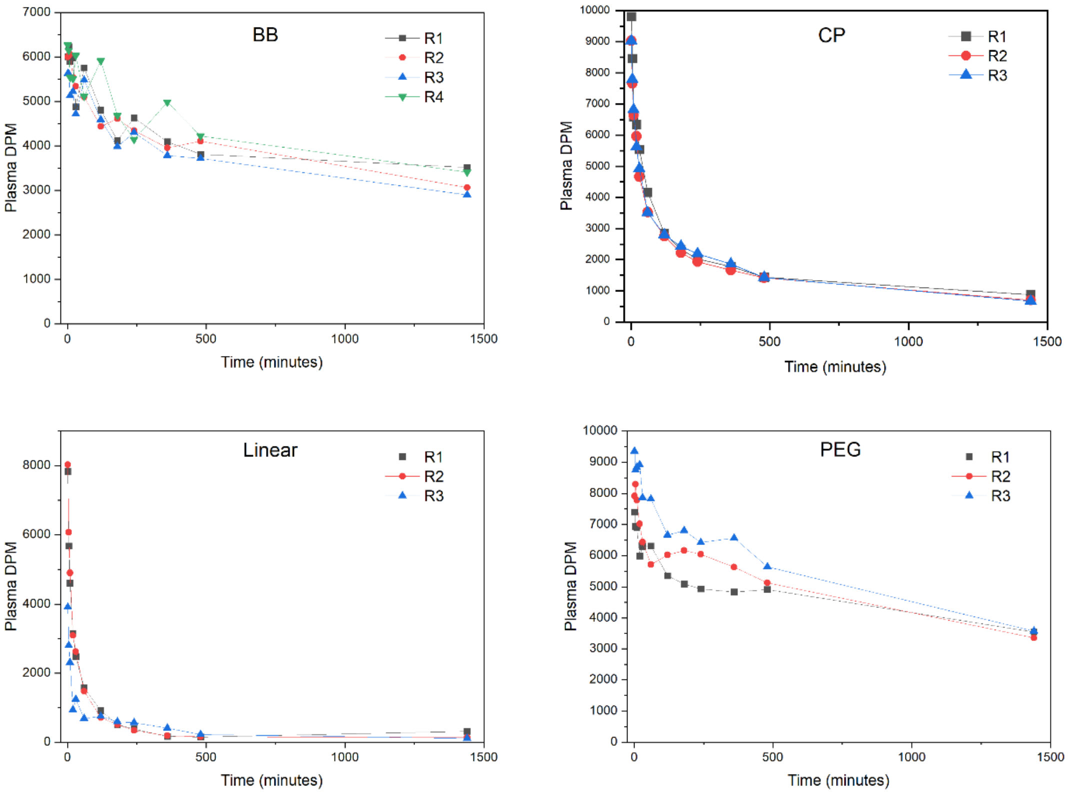

Figure S18: Uncorrected pharmacokinetic plasma concentrations of radiolabelled materials injected intravenously into rats (3-4 per compound).

References

1. Arnold, O.; Bilheux, J. C.; Borreguero, J. M.; Buts, A.; Campbell, S. I.; Chapon, L.; Doucet, M.; Draper, N.; Ferraz Leal, R.; Gigg, M. A.; Lynch, V. E.; Markvardsen, A.; Mikkelson, D. J.; Mikkelson, R. L.; Miller, R.; Palmen, K.; Parker, P.; Passos, G.; Perring, T. G.; Peterson, P. F.; Ren, S.; Reuter, M. A.; Savici, A. T.; Taylor, J. W.; Taylor, R. J.; Tolchenov, R.; Zhou, W.; Zikovsky, J., Mantid—Data analysis and visualization package for neutron scattering and SR experiments. *Nuclear Instruments and Methods in Physics Research Section A: Accelerators, Spectrometers, Detectors and Associated Equipment* **2014,** *764*, 156-166.

2. Heenan, R. K.; Rogers, S. E.; Turner, D.; Terry, A. E.; Treadgold, J.; King, S. M., Small Angle Neutron Scattering Using Sans2d. *Neutron News* **2011,** *22* (2), 19-21.

3. S. C. Larnaudie, J. C. Brendel, K. A. Jolliffe and S. Perrier, *J. Polym. Sci., Part A: Polym. Chem.*, 2016, **54**, 1003-1011.
